# *Lactobacillus paracasei* ZJUZ2-3 inhibits gastrointestinal tumors via the IAA-induced AHR/MTDH/NF-κB axis

**DOI:** 10.7150/ijbs.114602

**Published:** 2025-10-10

**Authors:** Rui Yang, Yan Yang, Wu Lin, Kaikai He, Dexi Bi, Lisong Teng

**Affiliations:** 1 Department of Surgical Oncology, The first Affiliated Hospital, School of Medicine, Zhejiang University, Hangzhou, 310003, China.; 2 Department of Colorectal Surgery and Oncology (Key Laboratory of Cancer Prevention and Intervention, China National Ministry of Education), 310003, China.; 3 The Second Affiliated Hospital, Zhejiang University School of Medicine, Hangzhou, Zhejiang, 310003, China.; 4 Department of Orthopaedics, the Second Affiliated Hospital of Xi'an Jiaotong University, Xi'an, 710000, China.; 5 Department of Pathology, Shanghai Tenth People's Hospital, Tongji University School of Medicine, Shanghai, 200072, China.

**Keywords:** gastrointestinal cancer, *L. paracasei*, indole-3-acetic acid, probiotic, novel species

## Abstract

*Lactobacillus paracasei* abundance is markedly reduced in gastric cancer (GC) tissues, suggesting its potential protective role. From healthy gastric tissue of a GC patient, we isolated a novel strain, *L. paracasei* ZJUZ2-3, which exerted potent antitumor effects. Intratumoral injection of live ZJUZ2-3, but not heat-killed bacteria, attenuated gastrointestinal tumor growth in mice. Conditioned medium from ZJUZ2-3 similarly inhibited cancer cell proliferation, implicating a secreted metabolite. Metabolomic profiling identified indole-3-acetic acid (IAA) as the key bioactive compound. Consistent with this, genetic knockout of *trpA* (essential for IAA biosynthesis) in ZJUZ2-3 abolished its antitumor efficacy. IAA alone recapitulated the tumor-suppressive effects both *in vitro* and *in vivo*. Mechanistically, IAA activated the aryl hydrocarbon receptor (AHR), which then competitively bound to metadherin (MTDH). This interaction blocked MTDH phosphorylation and the subsequent activation of NF-κB signaling. Crucially, depletion of either AHR or MTDH abrogated IAA's efficacy, underscoring the essential role of this axis. Furthermore, ZJUZ2-3 synergized with conventional chemotherapy, potentiating tumor regression. While this study lacks humanized immune models and exploration of strain-specificity, our findings identify *L. paracasei* ZJUZ2-3 and its effector metabolite IAA as promising precision microbiome-based therapeutics targeting the AHR-MTDH-NF-κB pathway for adjuvant treatment of GC.

## Introduction

Gastrointestinal (GI) malignancies, encompassing gastric cancer (GC), esophageal cancer (EC), and colorectal cancer (CRC), represent a significant global health burden as both highly prevalent and lethal neoplasms. Among these, gastric cancer ranks as the fifth most frequently diagnosed malignancy worldwide and remains one of the leading causes of cancer-associated mortality globally. 1. Although *Helicobacter pylori* is broadly recognized as a major risk factor for GC 2, emerging evidence suggests that non-*H. pylori* microbes and microbial dysbiosis contribute to carcinogenesis across the GI tract 3,4. The gut and gastric microbiota play pivotal roles in modulating inflammation, immune regulation, and metabolic homeostasis. However, the mechanistic contributions of specific bacterial species to gastrointestinal (GI) carcinogenesis remain incompletely understood. Notably, emerging evidence suggests that certain probiotic strains—such as *Lactobacillus plantarum*—exert anti-tumorigenic effects through immunomodulatory mechanisms, highlighting their potential as novel therapeutic adjuvants in GI oncology. 5.

The potential cancer-therapy effects of probiotics have been reported in experimental studies and clinical trials 6,7. Our previous work revealed a significant depletion of *Lactobacillus paracasei* in gastric cancer (GC) tumor tissues compared to adjacent normal tissues 8. Beyond its established role in promoting digestive health—through modulation of gut microbiota, maintenance of intestinal homeostasis, and immune enhancement—*L. paracasei* exhibits robust acid and bile salt tolerance, a critical feature for probiotic survival and functionality in the gastrointestinal tract 9. While certain strains, such as *L. paracasei* PS23, have demonstrated safety and efficacy in clinical applications 10, their precise mechanisms of action in GI cancer prevention remain poorly characterized. In particular, the functional contributions of *L. paracasei* to gastric carcinogenesis and its underlying molecular pathways require further elucidation.

The gut microbiota produces a diverse array of metabolites, including secondary bile acids, short-chain fatty acids (SCFAs), indole derivatives, and amino acid metabolites. 11. Our previous study has revealed a positive correlation between the indole-3-acetic (IAA) levels and *L. paracasei* abundance in gastric tissues 12. Indole-3-acetic acid (IAA), a key tryptophan (Trp) metabolite, functions as a physiological ligand for the aryl hydrocarbon receptor (AHR). Emerging evidence indicates that AHR activation modulates the nuclear factor kappa B (NF-κB) signaling pathway through molecular crosstalk.

In this study, we isolated and characterized a novel probiotic strain, *Lactobacillus paracasei* ZJUZ2-3, which demonstrated significant tumor-suppressive activity across multiple gastrointestinal cancer models. Furthermore, our investigation revealed that the observed antitumor effects were mediated by the microbial metabolite indole-3-acetic acid (IAA). Mechanistically, the microbial metabolite indole-3-acetic acid (IAA) exerts its antitumor effects by acting as an aryl hydrocarbon receptor (AHR) agonist. AHR activation inhibits MTDH phosphorylation at Ser298, which in turn suppresses nuclear factor kappa B (NF-κB) signaling pathway activity, ultimately attenuating gastric cancer progression. Notably, the antitumor efficacy of *L. paracasei* ZJUZ2-3 extended to both esophageal and colorectal cancer models, demonstrating broad-spectrum activity against gastrointestinal tumors. Collectively, our work not only identifies a novel *L. paracasei* strain with potent pan-GI anticancer properties but also elucidates its mechanistic basis through the IAA-AHR-NF-κB signaling axis.

## Results

### *L. paracasei* abundance was decreased in tumor tissue samples of GC patients

**Figure 1:**
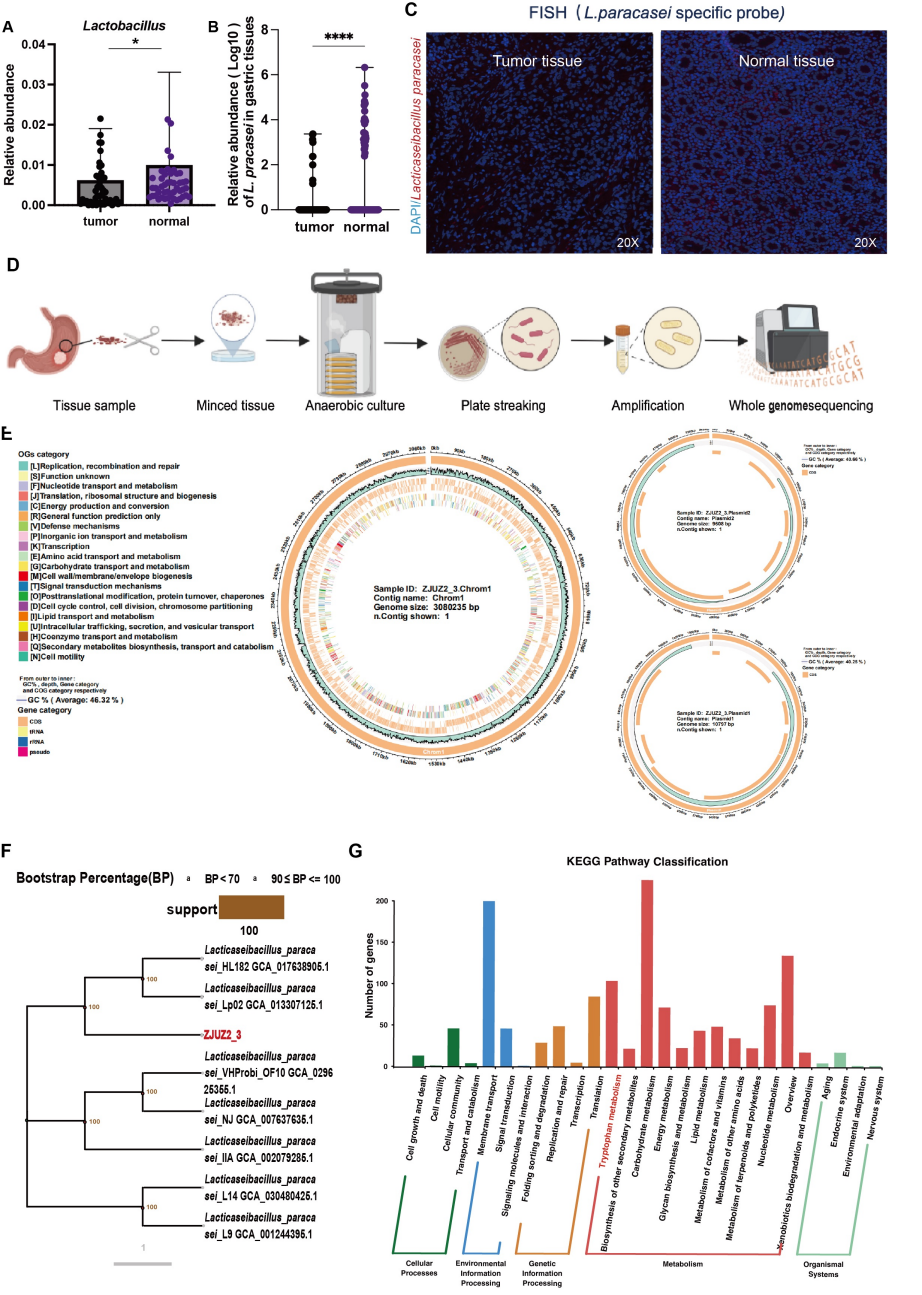
***L. paracasei* ZJUZ2-3 was isolated from a GC patient normal tissue.** (A) The abundance of *Lactobacillus* in patients with GC (N=49). (B) The relative abundance of *L. paracasei* in tumor tissues and normal tissues of GC patients. (C) Representative FISH images of gastric tissue sections from GC patients (blue: nuclear, green: *L.paracasei* probe); scale bars, 100 μm. (D) Isolation of a novel *L.paracasei* strain from a GC patient's normal tissue. (E) The whole genome map of *L.paracasei* ZJUZ2-3*.* The circle chart displays GC content, sequencing depth, gene element display, and COG function display from the outside to the inside. (F) Phylogenetic tree of *L.paracasei* ZJUZ2-3*.* A phylogenetic tree based on core single copy genes constructed using Neighbour joining clustering. (G) Functional annotation of *L.paracasei* ZJUZ2-3 genome using KEGG metabolic pathway. Data in A and B were analyzed by t tests; Means ± SD. ns ≧0.05, *p < 0.05, **p < 0.01, ***p < 0.001, ****p < 0.0001.

Probiotics are beneficial bacteria exerting health benefits once consumed. Multiple probiotics have been reported to exhibit inhibitory effect against GC through releasing specific proteins or metabolites. Here we validated the enrichment of *Lactobacillus* in normal gastric tissues with qPCR in paired tumor and adjacent normal tissues of 90 patients (Figure S1A). Then qPCR detection of major *Lactobacillus* species was also conducted, which revealed that *L. paracasei*, but not *L. johnsonii* or* L. reuteri*, had significantly higher relative abundance of this species in normal tissues than in tumor tissues (Figure 1A,1B, S1B & S1C). Interestingly, *L. paracasei* is a known probiotics with reported antitumor capability 13,14. The colonization of this species in the stomach was confirmed by fluorescence in situ hybridization (FISH) and qPCR detection of bacterial DNA (Figure 1C and Figure S1D). These findings suggest the potential protective role of *L.paracasei* in GC progression.

### A novel *Lactobacillus paracasei* ZJUZ2-3 was isolated from normal gastric tissue

We isolated a strain of *L. paracasei* (designated ZJUZ2-3) from the normal tissue of a gastric cancer patient (Figure 1D), and performed whole genome sequencing of the isolate. The complete genome of strain ZJUZ2-3 was determined and found to contain a circular chromosome with a size of 3,080,235 bp and a GC content of 46%. It contains 2,850 protein coding genes, 77 non-coding genes. Genomic testing revealed that the strain contains two plasmids with sizes of 9,535 bp and 10797 bp, respectively (Figure 1E). After software analysis, the whole genome ANIb value of the relative *Lactobacillus paracasei* strain (NCBI genome registration number: GCA_029625355.1) was 99.6656, and the whole genome ANIb value of the relative *Lactobacillus paracasei* strain (NCBI genome registration number: GCA_030480425.1) was 99.6618. ANIb, Average nucleotide identity using BLAST, also known as average nucleotide identity using BLAST, is an indicator for evaluating the phylogenetic relationship of strains. It is generally believed that ≥ 95 indicates belonging to the same species. The genome of the above-mentioned strain ZJUZ2-3 is not completely consistent with the genome of *Lactobacillus paracasei* in the NCBI database. The species evolution tree showed that ZJUZ2-3 was a subspecies of *Lactobacillus paracasei* (Figure 1F). Notably, it possessed a tryptophan metabolism pathway. We found the gene tryptophan synthase subunit alpha (TrpA) which can synthesize tryptophan (Figure 1G).

### *L.paracasei* ZJUZ2-3 and its conditioned medium inhibited GC progression *in vitro* and *in vivo*

**Figure 2:**
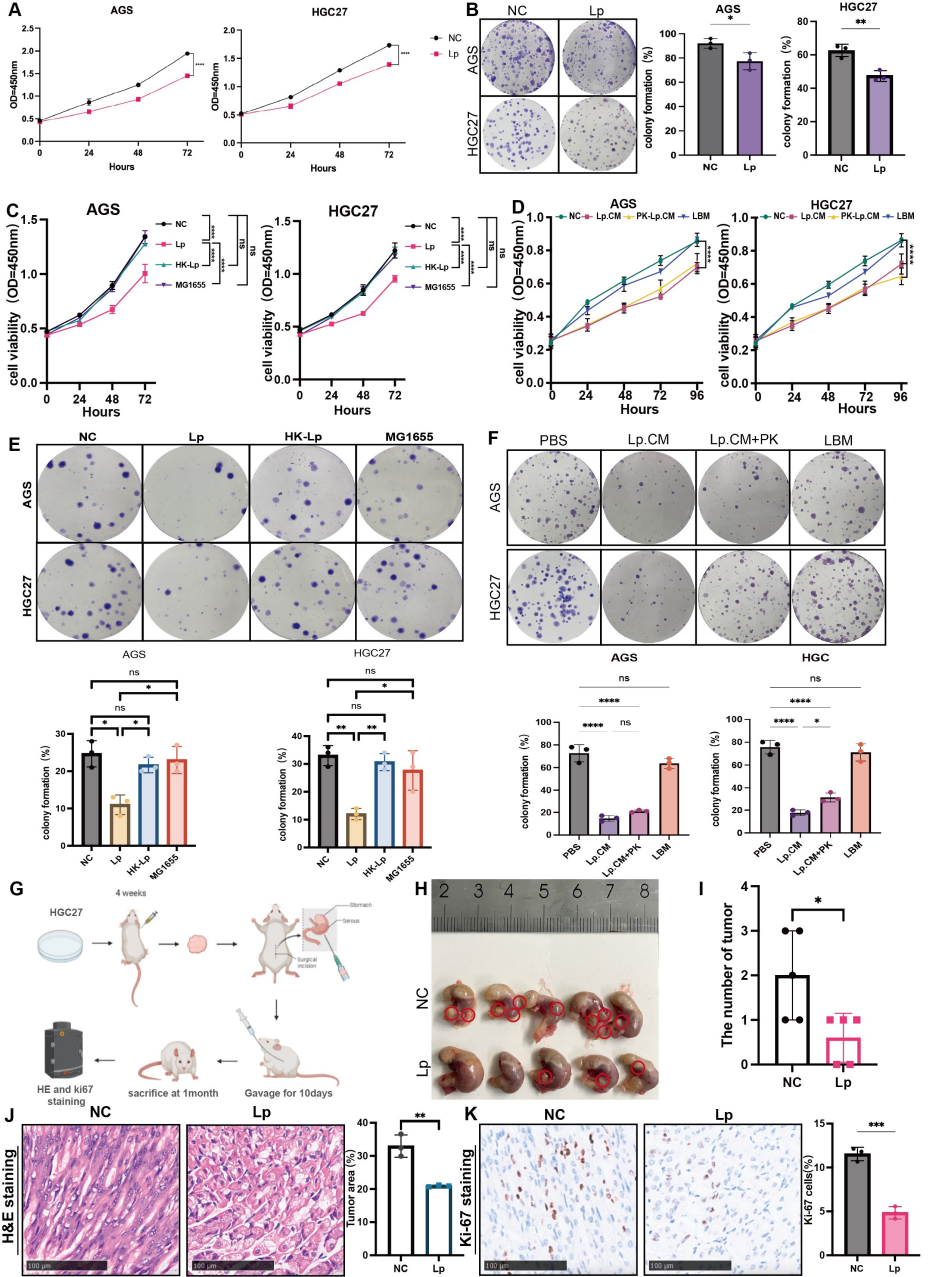
** Living *L. paracasei* ZJUZ2-3 and its metabolites could inhibit GC cell growth and tumor growth in the GC xenograft mice.** (A) *L.paracasei* ZJUZ2-3 (MOI=100) inhibits the cell viability of gastric cancer cells AGS and HGC27 as determined by CCK8 assays. (Lp: *L. paracasei* ZJUZ2-3) (B) Colony formation of GC cells under the treatment of* L.paracasei* ZJUZ2-3. (C) Cell viability at OD450nm of GC cells under the treatment of *L. paracasei* ZJUZ2-3, heat-killed *L. paracasei* ZJUZ2-3 and *E.coli* MG1655. (D) Cell viability at OD450nm of GC cells treated with PBS, *L. paracasei* conditional medium (Lp.CM), *L. paracasei* conditional medium with digestion by protease K (PK-Lp.CM) and lactobcillus broth medium (LBM). (E) Colony formation of GC cells under the treatment of *L. paracasei* ZJUZ2-3, heat-killed *L. paracasei* ZJUZ2-3 and *E.coli* MG1655. (F) Colony formation of GC cells treated with PBS, Lp.CM, PK-Lp.CM and LBM. (G) Schematic diagram shows the experimental design, time line and treatment of the GC orthotopic tumor model (Lp: *L. paracasei* ZJUZ2-3). (H) The representative stomach morphologies of mouse. (I) The number of orthotopic tumors. (J) Representative HE staining images of tumor tissues from mice administrated with PBS and *L. paracasei* ZJUZ2-3. (K) Ki-67 staining showed decreased numbers of Ki-67^+^ cells in the tumor tissues of *L. paracasei* ZJUZ2-3 treated groups. Data in A, C, D, E and F were analyzed by two-way ANOVA, Data in B, I and K were analyzed by t tests; Means ± SD. ns ≧0.05, *p < 0.05, **p < 0.01, ***p < 0.001, ****p < 0.0001.

We next determined whether *L. paracasei* ZJUZ2-3 had anti-tumor effect *in vitro*. Gastric cancer cell lines AGS and HGC27 were co-cultured with *L. paracasei* ZJUZ2-3 (1×10^7^ CFU/ml) for 4 hours, while PBS was used as blank control. *L. paracasei* co-culture significantly decreased GC cell viability (Figure 2A). This growth-inhibitory effect was further confirmed by colony formation assay (Figure 2B). Live *L. paracasei* ZJUZ2-3 was heat-killed by autoclaving and subsequently exposed to gastric cancer cells. The *Escherichia coli (E. coli)* strain MG1655 was used as bacteria control. In contrast, neither heat-killed *L. paracasei* ZJUZ2-3 (HK-Lp) nor *E. coli* MG1655 induced the reduction of gastric cancer cell viability (Figure 2C and E).

We next explored whether such function was attributed to *L. paracasei* ZJUZ2-3 itself or its metabolites. *L. paracasei* culture was centrifuged and the supernatant was then filtered through a 0.22 µm membrane to obtain a conditional culture medium (CM), which was used to treat AGS and HGC27 cells at the concentration of 1% (v/v), 5% (v/v) and 10% (v/v). It was demonstrated that *L.paracasei* conditonal culture medium (Lp.CM) inhibited tumor cell proliferation in a dose-dependent manner. (Figure S2A and S2B). Therefore, we treated gastric cancer cells with 1% (v/v) *L. paracasei* conditioned medium (Lp. CM), using phosphate-buffered saline (PBS) and lactobacillus basal medium (LBM) as negative control. Interestingly, the CM significantly inhibited AGS and HGC27 growth (Figure 2D and 2F). To further distinguish the type of molecules responsible for such effect, the *L. paracasei* conditional medium (Lp.CM) was pretreated with or without protease K (PK, 1 mg/mL). The inhibitory effect remained unchanged after PK treatment, indicating that the antitumor effect was dependent on non-protein metabolites in the Lp.CM (Figure 2D and 2F).

Mouse experiments were further conducted to assess the potential protective effect of *L. paracasei* ZJUZ2-3 against GC. We then assessed the efficacy of *L. paracasei* ZJUZ2-3 in an orthotopic mouse model of GC (Figure 2G). Nude mice bearing orthotopic HGC27(3×10^6^ cells). They were oral gavaged with PBS or *L. paracasei* ZJUZ2-3 at a dose of 1×10^8^CFU/100μl, for 10days. As shown in Figure 2H and 2I, oral gavage of *L. paracasei* ZJUZ2-3 significantly suppressed tumor progression in mice Histopathological (HE) and Ki-67 immunohistochemical analyses demonstrated *L. paracasei* ZJUZ2-3 inhibited the orthotopic tumor growth (Figure 2J and K).

Since probiotics could confer benefits against gastric tumourigenesis by modulating microbiota, we elucidated the impact of *L. paracasei* ZJUZ2-3 on the gut microbiota by performing 16s rRNA sequencing on stomach samples of ortho topic tumor mice. β-Diversity analysis by PCoA showed that the microbial community in *L. paracasei* ZJUZ2-3-treated mice was distinctively separated from the control group (Figure S1F), implying that *L. paracasei* ZJUZ2-3 could alter the composition of gut microbiota. A significant shift of microbial species was also observed in *L. paracasei* ZJUZ2-3-treated mice, particularly including the enrichment of known probiotics such as *Lactobacillus johnsonii*, *Lactobacillus_paracsei* and *Bifidobacterium_longum*. These findings therefore implied that* L. paracasei* ZJUZ2-3 could elevate the abundance of commensal probiotics against GC tumourigenesis in mice. In addition, differential analysis identified the enrichment of L. paracasei ZJUZ2-3 in gavaged-treated mice (Figure S1G). Those results confirmed the protective effect of *L. paracasei* ZJUZ2-3 against gastric tumorigenesis.

### Protective effect of *L.paracasei* ZJUZ2-3 is associated with the production of indole-3-acetic acid (IAA)

**Figure 3:**
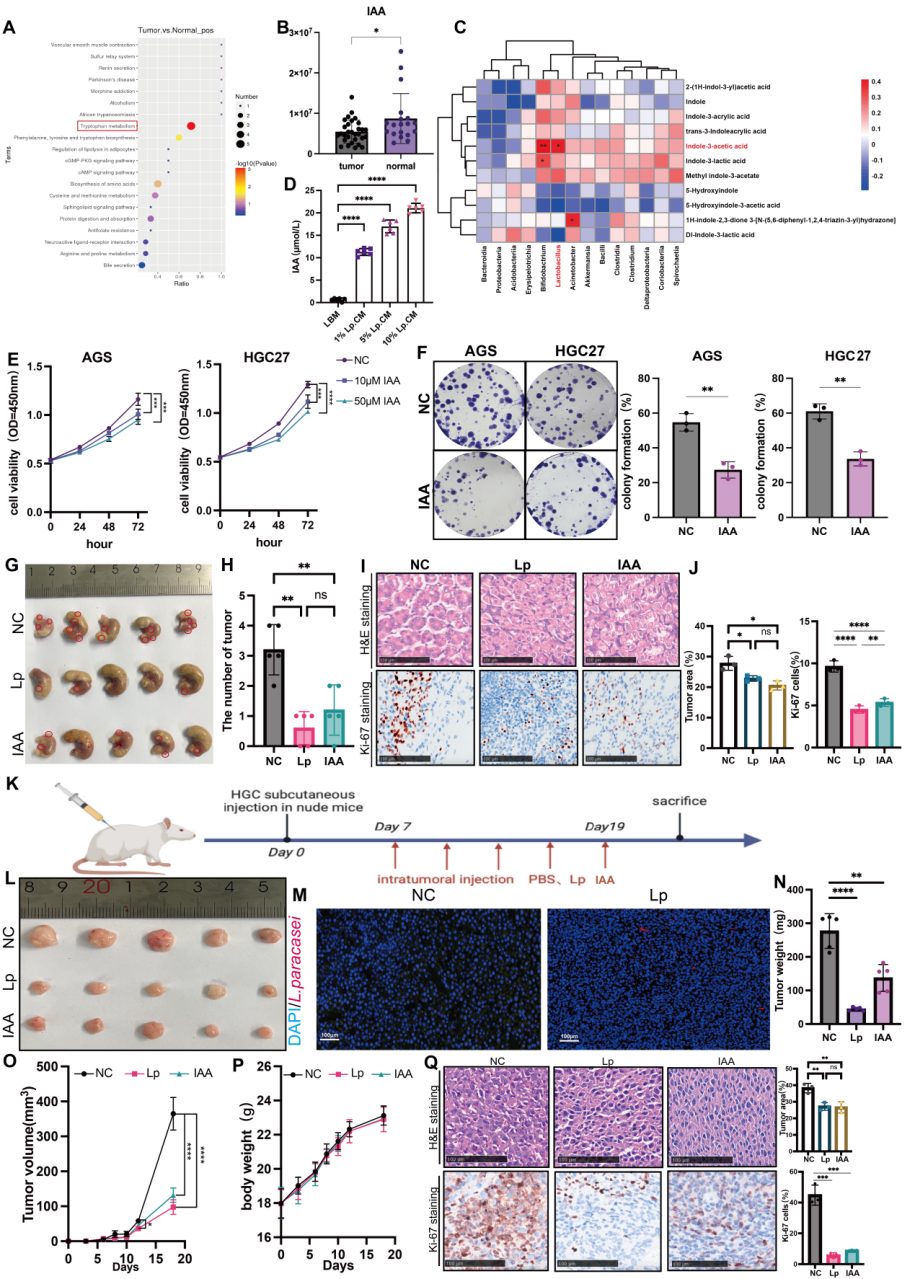
** IAA inhibited tumor cell growth *in vitro* and *in vivo*.** (A) KEGG pathway enrichment analysis of DEGs from RNA sequencing dataset of GC patients. Size of circles represents the ratio of DEGs in pathways. Red: upregulated, blue: downregulated. (B) The abundance of IAA in GC patients. (C) Correlation analysis between bacteria and indole derivatives. (D) The concentration of IAA in GC cells supernatant co-cultured with *L. paracasei* ZJUZ2-3 were detected by ELISA. (E) Cell viability at OD450nm of human GC cells (AGS, HGC27) treated with PBS or IAA. (F) Colony formation of GC cells under treatment of PBS or IAA (10 μM). (G) The representative stomach morphologies of mouse. (H) The number of orthotopic tumor. (I) Representative HE and Ki-67 staining images of tumor tissues from mice administrated with PBS, *L. paracasei* ZJUZ2-3 and IAA. (J) Proportion of tumor area in HE staining images and Ki-67 staining showed decreased numbers of Ki-67^+^ cells in the tumor tissues of IAA and *L. paracasei* ZJUZ2-3 treated groups. (K) Schematic diagram shows the experimental design, time line and treatment of the GC xenograft mice (Lp: *L. paracasei* ZJUZ2-3). (L) The representative tumor morphologies. (M) *L. paracasei* ZJUZ2-3 colonization was confirmed by FISH. (N) Tumor weight compared between IAA-, *L. paracasei* ZJUZ2-3- or PBS-treated groups. (O) Tumor growth curve of IAA-, *L. paracasei* ZJUZ2-3- and PBS-treated groups. (P) The mouse body weight curve of IAA-, *L. paracasei* ZJUZ2-3- and PBS-treated groups. (Q) Representative H&E image of tumor tissues from mice administrated with PBS, IAA and *L. paracasei* ZJUZ2-3. Data in D, E, J, N, O and Q were analyzed by two-way ANOVA, Data in B, F and H were analyzed by t tests; Means ± SD. ns ≧0.05, *p < 0.05, **p < 0.01, ***p < 0.001, ****p < 0.0001.

*L. paracasei* has been shown to release metabolites, including the indole-derivative indole-3-acetic acid (IAA) to interact with the host 15. Based on our previous untargeted metabolome analysis, we found that discriminative metabolites in tumor tissue and normal tissues of gastric cancer patients were enriched in the tryptophan pathway (Figure 3A) 8. The tryptophan metabolite IAA was the most significantly enriched in normal tissues (Figure 3B). Correlation analysis revealed that *Lactobacillus* abundance was positively correlated with IAA, suggesting that IAA might play a role in the inhibitory tumor progression of *L.paracasei* ZJUZ2-3 (Figure 3C). We subsequently employed ELISA to quantify the concentration of IAA in Lp.CM at various volume ratios (1%, 5%, and 10% v/v). Our results indicated that the IAA concentration exhibited a dose-dependent trend in Lp.CM, which provided a critical foundation for determining the subsequent IAA treatment concentrations (Figure 3D).

Taken together, these data demonstrate that the antitumor effect of *L. paracasei* ZJUZ2-3 was likely mediated by IAA. Upon establishing the involvement of IAA in *L. paracasei*-mediated tumor suppression, we assessed whether IAA alone is sufficient to induce an antitumor response. Remarkably, IAA significantly inhibited the viability of AGS (*p*<0.01) and HGC27 (*p*<0.01) cells in a concentration-dependent manner (Figure 3E). IAA treatment also markedly restrained the colony formation of GC cells, compared with control (*p*<0.01; Figure 3F).

Subsequently, we verified the impact of *L.paracasei* ZJUZ2-3 and IAA on the nude mouse xenograft model. Four-week-old nude mice were subcutaneously injected with HGC27 cells (3×10^6^ cells). *L. paracasei* ZJUZ2-3 (1×10^8^ CFU/100 μl) or IAA (100µM, 100µl) or PBS (100µl) with an equivalent volume (blank control) was intratumorally injected once in two days (Figure 3K). After seven days, *L. paracasei* ZJUZ2-3- and IAA-treated mice had an obvious reduction in tumor size and weight compared to those injected with PBS (Figure 3L, N and O). HE results indicated *L. paracasei* ZJUZ2-3 and IAA treated mice had a lower proportion of tumor area as compared with control (Figure 3Q)*. L. paracasei* ZJUZ2-3 and treatment markedly reduced the number of Ki-67-positive cells in tumor tissues (Figure 3Q). Successful intratumor colonization of *L. paracasei* ZJUZ2-3 was confirmed by FISH (Figure 3M) and qPCR with customized primers (Supplementary Figure 1E)*.* Compared with the control group, treatment with *L. paracasei* ZJUZ2-3 and IAA did not significantly affect body weight in nude mice.

**Figure 4:**
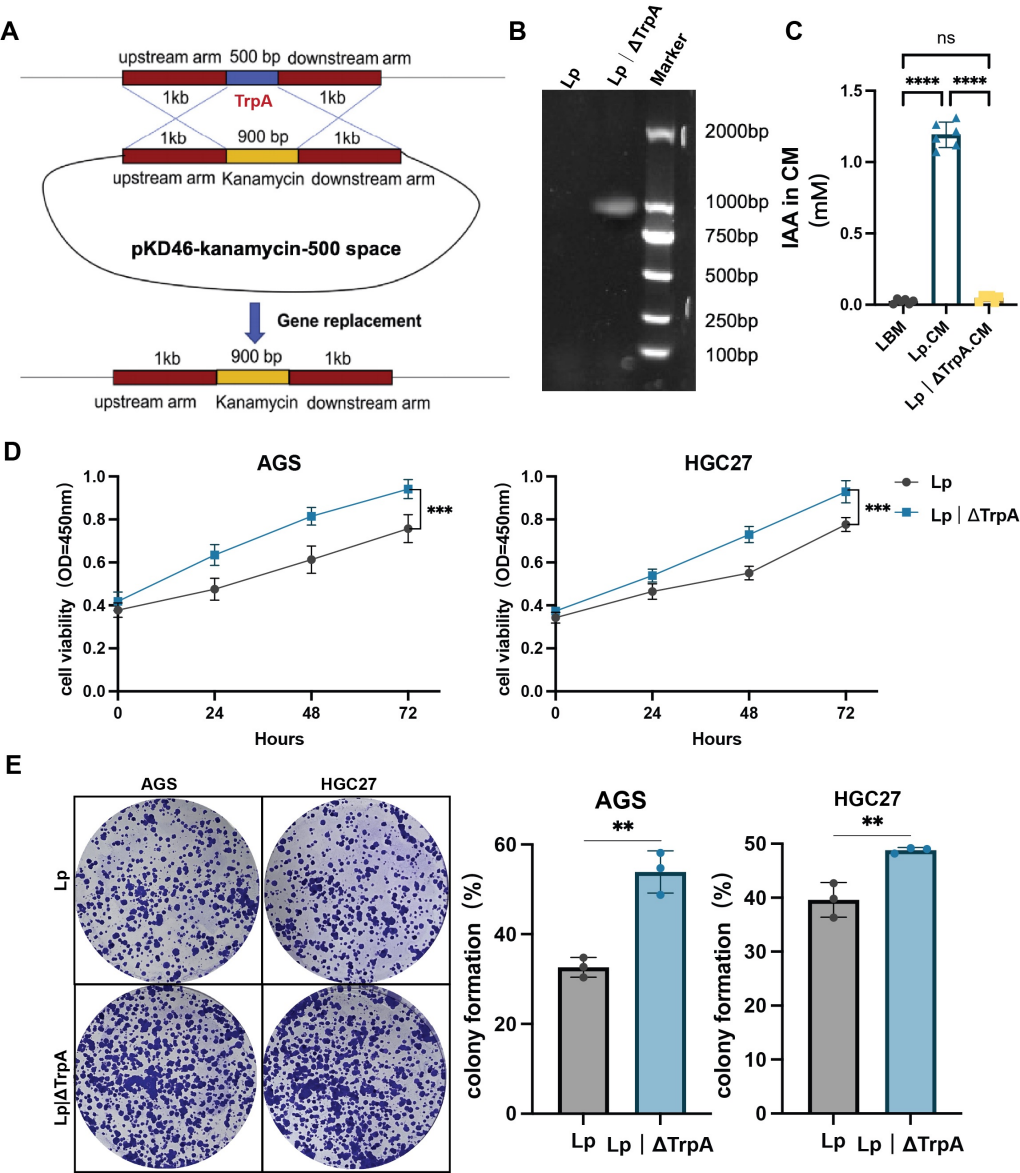
** Loss of antitumor activity in IAA-deficient *L. paracasei* ZJUZ2-3 mutants.** (A) Schematic diagram showing the construction of Lp|ΔTrpA mutant strain. (B) PCR validated the mutant and confirmed the successful replacement of *trpA* sequence into kanamycin resistance (*kan*^R^) cassette. (C) The concentration of IAA in LBM, Lp.CM and Lp|ΔTrpA CM. (D) Cell viability at OD450nm; (E) colony formation of human GC cells with Lp and Lp|ΔTrpA. (F) Lp,* L.paracasei.* Data in C and D were analyzed by two-way ANOVA, Data in E were analyzed by t tests; Means ± SD. **p < 0.01, ***p < 0.001, ****p < 0.0001.

TrpA is an essential enzyme for indole-3-acetic acid (IAA) biosynthesis in *Lactobacillus paracasei*. Consequently, we generated an isogenic trpA knockout mutant (Lp|ΔTrpA) using homologous recombination (Figure 4A and B). We quantified IAA levels by ELISA. The mutant strain produced significantly less IAA than the wild-type *L. paracasei* ZJUZ2-3 (Figure 4D and E).

### IAA mediates antitumor effects via activation of the aryl hydrocarbon receptor (AHR) and inhibits the activation of NF-κB signaling pathway

**Figure 5:**
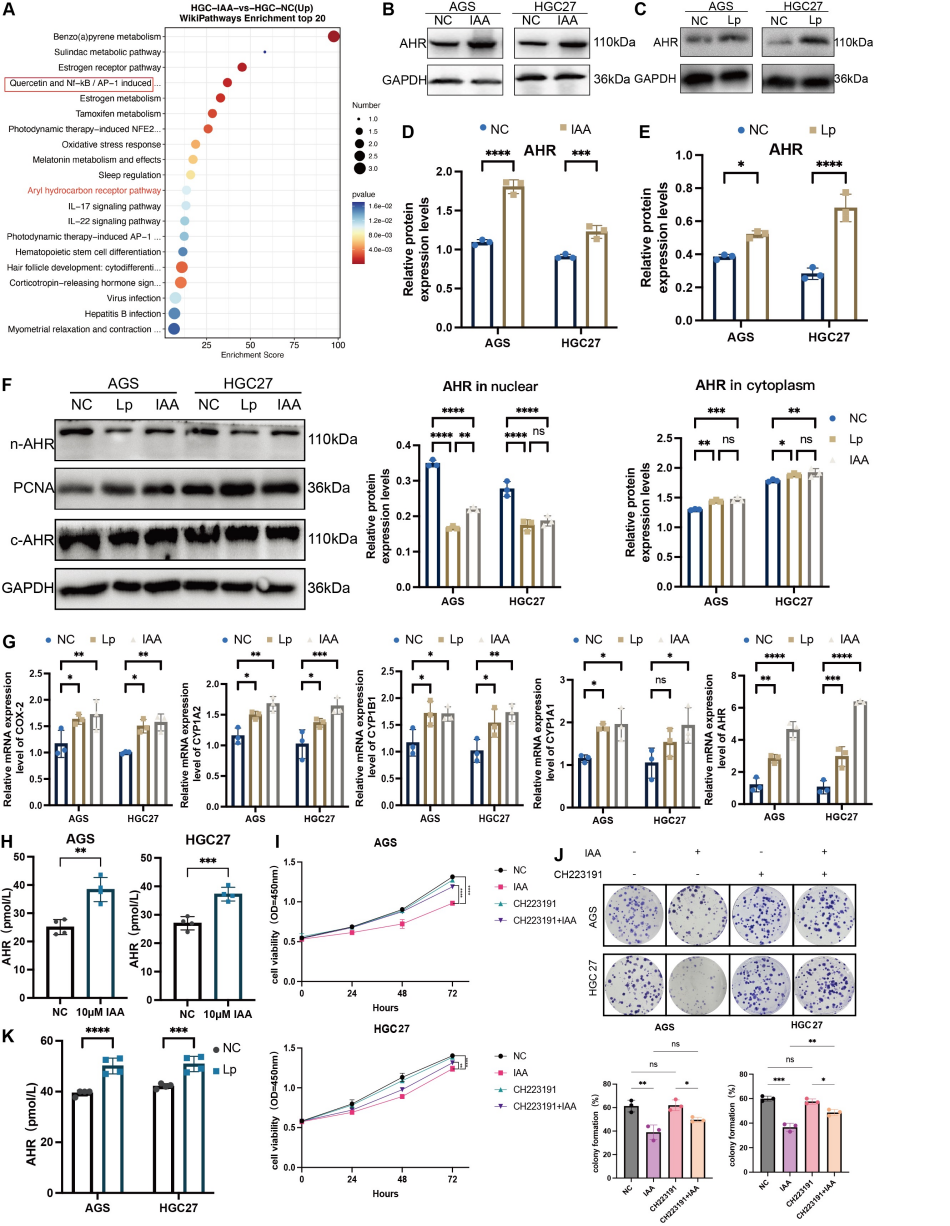
***L. paracasei* ZJUZ2-3 and IAA could upregulate AHR, and AHR antagonists could abolish the antitumor effect of IAA.** (A) Wiki pathway enrichment analysis of DEGs from RNA sequencing dataset of HGC27 cells treated with IAA. Size of circles represents the ratio of DEGs in pathways. Red: upregulated, blue: downregulated. (B, C) Western blot analysis of the activation of AHR in (B) IAA- and (C) *L. paracasei* ZJUZ2-3*-* treated GC cells. (D, E) The relative protein expression levels of AHR in AGS and HGC27. Data were analyzed by t tests; Means ± SD. *p < 0.05, **p < 0.01, ***p < 0.001, ****p < 0.0001. (F) Western blot analysis of the activation of AHR in nuclear and cytoplasm in IAA- and *L. paracasei* ZJUZ2-3*-* treated GC cells. Data were analyzed by two-way ANOVA; Means ± SD. ns ≧0.05, *p < 0.05, **p < 0.01, ***p < 0.001, ****p < 0.0001. (G) The relative mRNA expression levels of AHR and its downstream pathway genes. Data were analyzed by two-way ANOVA; Means ± SD. ns ≧0.05, *p < 0.05, **p < 0.01, ***p < 0.001, ****p < 0.0001. (H) The concentration of AHR in GC cells co-cultured with (D)IAA (10 μM) and (E) *L. paracasei* ZJUZ2-3*.* Data were analyzed by t tests; Means ± SD. ns ≧0.05, *p < 0.05, **p < 0.01, ***p < 0.001, ****p < 0.0001. (I) Cell viability at OD450nm and (J)colony formation of human GC cells (AGS and HGC27) with PBS, IAA, CH223191(10μM), CH223191+IAA. Data were analyzed by two-way ANOVA; Means ± SD. ns ≧0.05, *p < 0.05, **p < 0.01, ***p < 0.001, ****p < 0.0001. (J) The concentration of AHR in GC cells co-cultured with *L. paracasei* ZJUZ2-3. Data were analyzed by two-way ANOVA; Means ± SD. ***p < 0.001, ****p < 0.0001. Data in F, G, I, and J were analyzed by two-way ANOVA, Data in D, E and H were analyzed by t tests; Means ± SD. ns ≧0.05, *p < 0.05, **p < 0.01, ***p < 0.001, ****p < 0.0001.

To investigate the mechanism triggered by IAA for GC inhibition, RNA sequencing was performed to profile gene expression alterations in GC cell treated with IAA, by which 100 deregulated genes were identified. Of note, the aryl hydrocarbon receptor (AHR) was significantly upregulated (Figure 5A). AHR, a DNA binding protein existing in the cytoplasm as an inactive state AHR signal, is highly expressed in GC cells, so AHR is effective only activated by its ligand 16. We further confirmed IAA treatment increased the protein levels (Figure 5B and D) and the activity (Figure 5H) of AHR in both GC cell lines. Accordingly, treatment with *L. paracasei* ZJUZ2-3 had the similar effect (Figure 5C, E and K). Surprisingly, we found that the AHR protein levels in the nuclear of the Lp and IAA groups were not increased as previously reported, and no significant difference was observed in the cytoplasm (Figure 5F). These observations reveal that AHR functions through an alternative, non-traditional mechanism following its nuclear translocation, rather than through conventional pathway activation. To validate our hypothesis regarding AHR's non-canonical activity, we quantified the expression of classical AHR target genes (CYP1A1, CYP1A2, CYP1B1, and COX2) following nuclear translocation via qPCR (Figure 5G). While *L. paracasei* and IAA treatment induced modest upregulation of these genes, the magnitude of induction was significantly attenuated compared to literature-reported levels for canonical AHR activation 17. These data suggest that AHR-mediated tumor suppression in gastric cancer operates independently of its traditional transcriptional paradigm, implicating an alternative mechanism downstream of nuclear localization.

**Figure 6:**
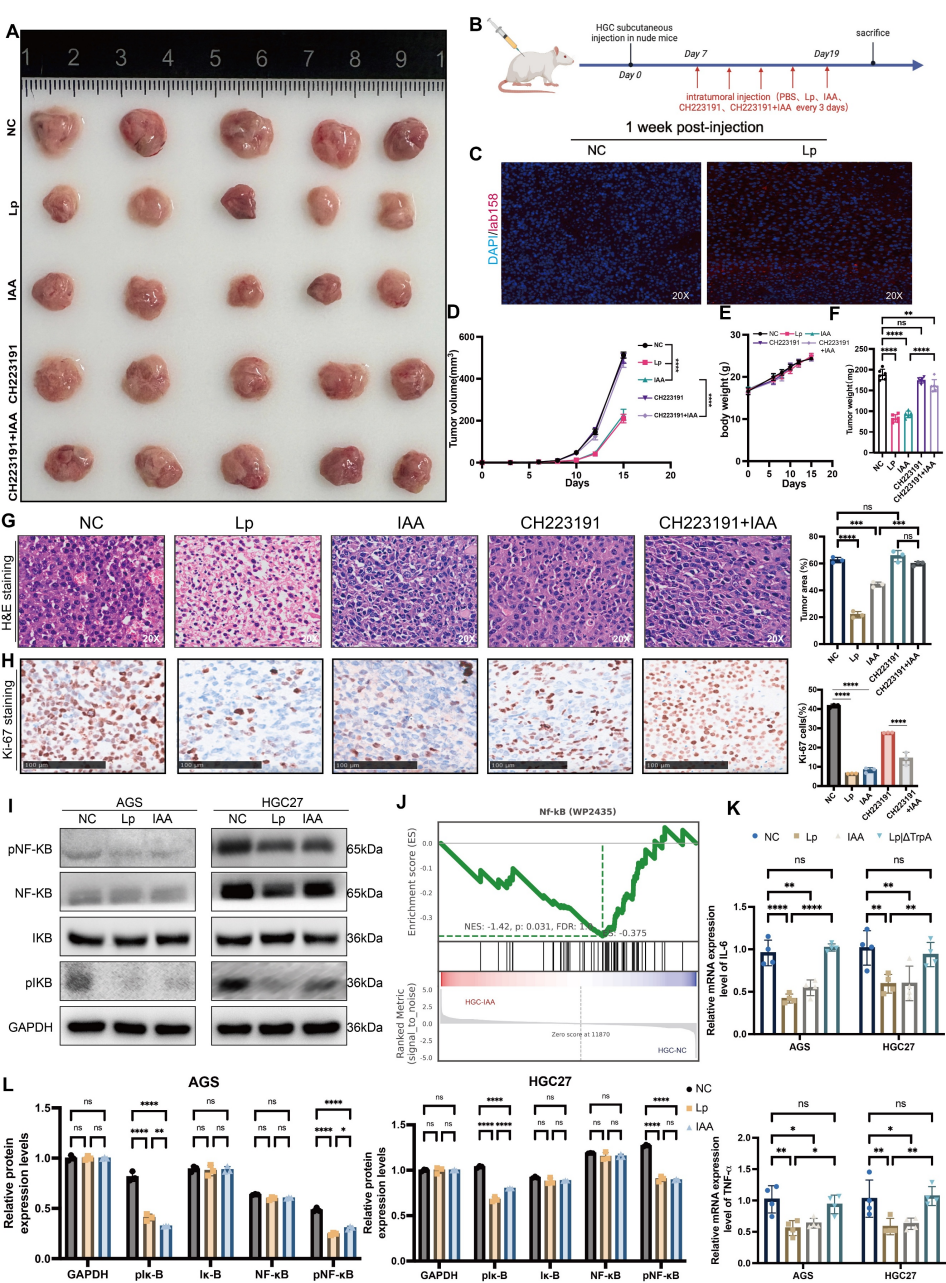
** IAA inhibit NF-kB signaling pathway through AHR. The representative tumor and mice morphologies.** (A) Schematic diagram shows the experimental design, time line and treatment of the GC xenograft mice. (B) Representative FISH images of tumor tissues from PBS- and *L. paracasei*,-injected mice. (blue: nuclear, green: FITC, red: *L. paracasei* probe); scale bars, 100 μm. (C) Tumor growth curve of mice from mice injected PBS, IAA, CH223191 and CH223191+IAA. (D) The body weight curve of HGC27 xenograft injected with PBS, *L. paracasei*, IAA, CH223191 and CH223191+IAA. (E) Tumor weight of HGC27 xenograft. (F) Representative images of H&E staining of tumor tissues of HGC27 xenograft injected PBS, IAA, CH223191 and CH223191+IAA. scale bars, 100μm. (G) Ki-67 staining showed decreased proliferation rates in *L. paracasei* and IAA injected mice compaired with PBS, CH223191 and CH223191+IAA. scale bars, 100μm. Lp, *L. paracasei.* (H) Western blot analysis of the inhibition of NF-kB of AGS and HGC27 treated with *L. paracasei*, and IAA. (I) Gene set enrichment analysis (GSEA) showing the enrichment of NF-kB signaling pathways. (J) The relative mRNA expression levels of IL-6 and TNF-ɑ in GC cells treated with PBS, *L. paracasei*, IAA (10μM) and Lp|ΔTrpA. (K) The relative protein expression levels of NF-kB signaling pathway. Lp, *L. paracasei.* Data in D, F, H, K and L were analyzed by two-way ANOVA; Means ± SD. ns *p*≧0.05, *p < 0.05, **p < 0.01, ***p < 0.001, ****p < 0.0001.

To investigate the direct link between the AHR pathway and the tumor-suppressive effect of *L. paracasei* ZJUZ2-3 and IAA, we used an AHR antagonist, CH223191, to block AHR activity. We observed that CH223191 treatment inhibit the antitumor effect of IAA via the CCK8 assay and colony formation test (Figure 5I and J). Consistently, intratumoral injection of CH223191 abolished IAA-induced tumor suppression *in vivo* (Figure 6A, B, D and F), these treatments did not impact the body weight of mouse (Figure 6E). HE staining images showed the proportion of tumor area (Figure 6G) and the ratio of Ki-67-positive cells was exhibited (Figure 6H).

Successful colonization of *L. paracasei* in tumor tissues of nude mice was also confirmed by FISH and PCR (Figure 6C and Figure S1E). Meanwhile, knockdown of *ahr* in GC cells also abrogated the protective function of IAA *in vitro* (Figure S2C-F). These findings demonstrated a critical role of AHR in IAA-mediated the antitumor effect in GC.

Numerous studies reported that the activation of AHR could inhibit the NF-κB signaling pathway, decrease the transcription of proinflammatory factors, including TNF-α and IL-6, and promote the release of anti-inflammatory factor IL-10, to prevent the proinflammatory process 18. Pathway enrichment analysis identified that several oncogenic pathways including NF-κB, IL-17, and IL-22 pathway were downregulated in IAA-treated HGC27 (Figure 5A). Enrichment score showed that NF-κB signaling was significantly inhibited by IAA (Figure 6J). Indeed, protein expression level of pNF-κB and pIKB-ɑ were decreased in *L. paracasei* ZJUZ2-3-infected and IAA treated GC cells as shown by western blot (Figure 6I). Moreover, qPCR analysis revealed significant downregulation of NF-κB downstream effector genes (IL-6, TNF-ɑ) in cells treated with either *L. paracasei* or IAA, compared to controls (p < 0.01, two-way ANOVA). In contrast, the Lp|ΔtrpA mutant strain (lacking IAA production) failed to suppress these pro-inflammatory cytokines, with expression levels comparable to controls (Figure 6K). These results functionally link IAA-dependent AHR activation to NF-κB pathway inhibition. Our results collectively indicated that IAA can downregulate NF-κB signaling pathway, which in turn contributes to its antitumor effect in GC.

### IAA suppressed NF-κB signaling pathway through inhibiting IKB-mediated MTDH phosphorylation

**Figure 7:**
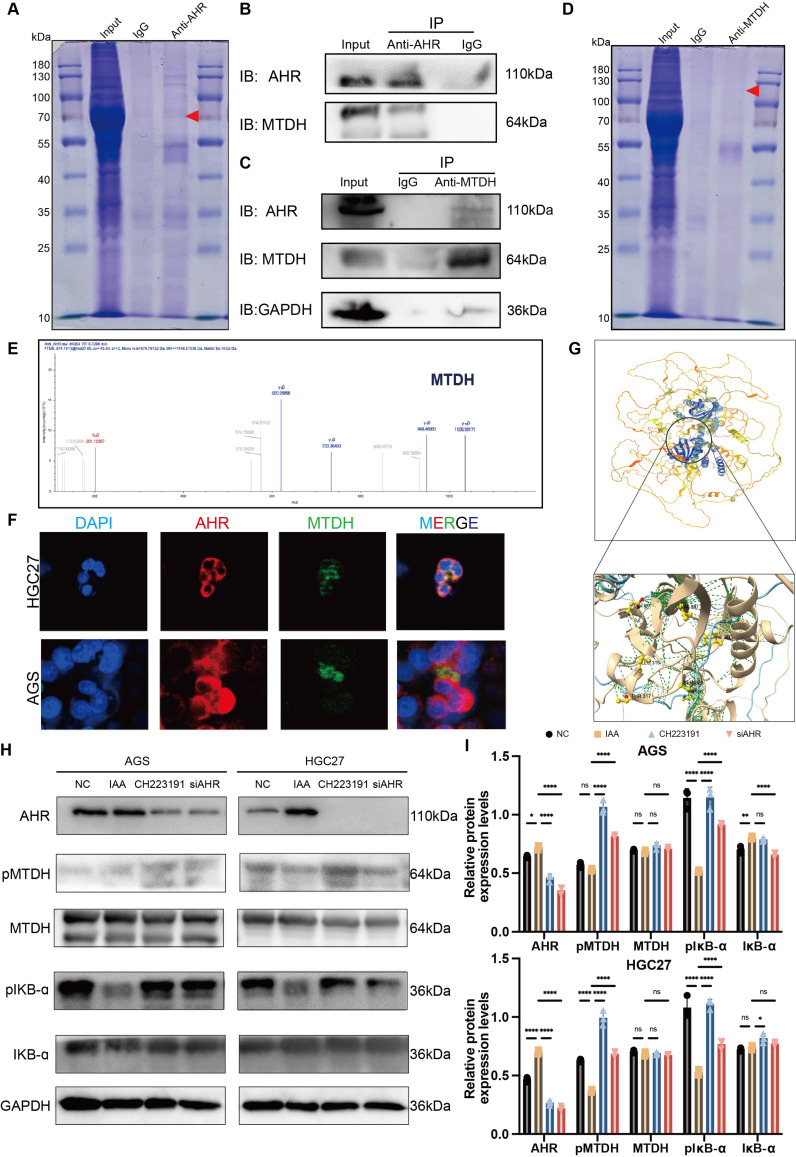
** AHR complete with IKB-ɑ binding MTDH to suppress NF-kB activation.** (A) Co-immunoprecipitation (Co-IP) assays showed the protein binding with AHR. (B) Immunoprecipitation of AHR and MTDH HGC27 cells lysates also showed positive binding between AHR and MTDH. (C) Immunoprecipitation of GAPDH, AHR and MTDH HGC27 cells lysates also showed positive binding between AHR and MTDH. (D) Co-immunoprecipitation (Co-IP) assays showed the protein binding with MTDH. (E) Mass spectrometry analysis demonstrated the protein MTDH. (F) Immunofluorescence (IF) used to confirm the binding of AHR and MTDH. (G) The protein-ligand interaction complex was generated by HDock. ChimeraX was used to visualize the AHR-MTDH complex and binding sites were displayed. (H) Western blot analysis of the inhibition of NF-kB signaling pathway through suppressing the phosphorylation of MTDH. (I) The relative protein expression levels of AGS and HGC27. Data were analyzed by two-way ANOVA; Means ± SD. ns *p*≧0.05, *p < 0.05, **p < 0.01, ***p < 0.001, ****p < 0.0001.

To determine if AHR suppresses NF-κB activation in gastric cancer (GC) cells, we performed Co-IP assays in HGC27 cells (Figure 7A). AHR-associated proteins were isolated and analyzed by LC-MS/MS (Figure 7E). We identified >10 candidate proteins, including known AHR binders HSPA7 and VIM (Table S1). Notably, no NF-κB subunits were detected. This suggests AHR does not directly interact with NF-κB 20 21. However, the oncoprotein Metadherin (MTDH), which binds to IKK-B to promote NF-kB activity in chemical HCC 22, was identified as a putative NF-kB-associated protein among the candidates (Figure 7B). To further validate the direct interaction between AHR and MTDH, we performed a reciprocal co-immunoprecipitation (Co-IP) assay (Figure 7D). Specifically, immunoprecipitation was carried out using an MTDH-specific antibody, followed by Western blot analysis to detect AHR protein expression. The results clearly demonstrated the presence of AHR protein bands in the MTDH immunoprecipitated complexes (Figure 7C), providing direct evidence for a specific interaction between AHR and MTDH. Immunofluorescence results showed the co-localization of AHR and MTDH in GC cells, further proving the interaction between AHR and MTDH (Figure 7F).

The interaction between MTDH and IKK-B promotes NF-kB nuclear translocation induced by the tumor necrosis factor α (TNF-α) 23. MTDH can be phosphorylated by IKK at Ser297 (murine) or Ser298 (human), and only phosphorylated MTDH acts as a co-activator for NF-kB on some of its target gene promoters 23,24. Therefore, we speculated that IAA could activate AHR, and then inhibit IKK-mediated MTDH phosphorylation to suppress NF-κB signaling pathway. We thus determined whether AHR affected the interaction between MTDH and IKB-ɑ in GC cells. MTDH phosphorylation (pMTDH) and IKB-ɑ phosphorylation (pIKB-ɑ) was increased in GC cells treated with siAHR or CH223191 (Figure 7H and I). Conversely, IAA-treated groups alleviated the phosphorylation of MTDH and IKB-ɑ. Thus, AHR could inhibit IKB-mediated MTDH phosphorylation, thereby suppressing GC cells NF-kB activation.

To explore the direct binding between AHR and MTDH, HDock was employed to predict protein-protein interaction molecular docking (Figure 7G). The analysis of protein-protein interaction surfaces based on PDBePISA indicated that GLY237 and GLY240 were putative binding sites for AHR and MTDH (Figure S3A, B and C). Collectively, AHR inhibits MTDH phosphorylation by IKB and the inhibitory of AHR relieves this inhibition, thereby preventing MTDH to promote GC cells NF-kB activation.

Overall, these results suggested that IAA promotes the transcriptional expression of AHR, which may bind to MTDH to inhibit its phosphorylation, thereby suppressing the activation of the NF-κB signaling pathway.

### *L. paracasei* ZJUZ2-3 inhibits gastrointestinal tumor growth and enhances chemotherapy efficacy

**Figure 8:**
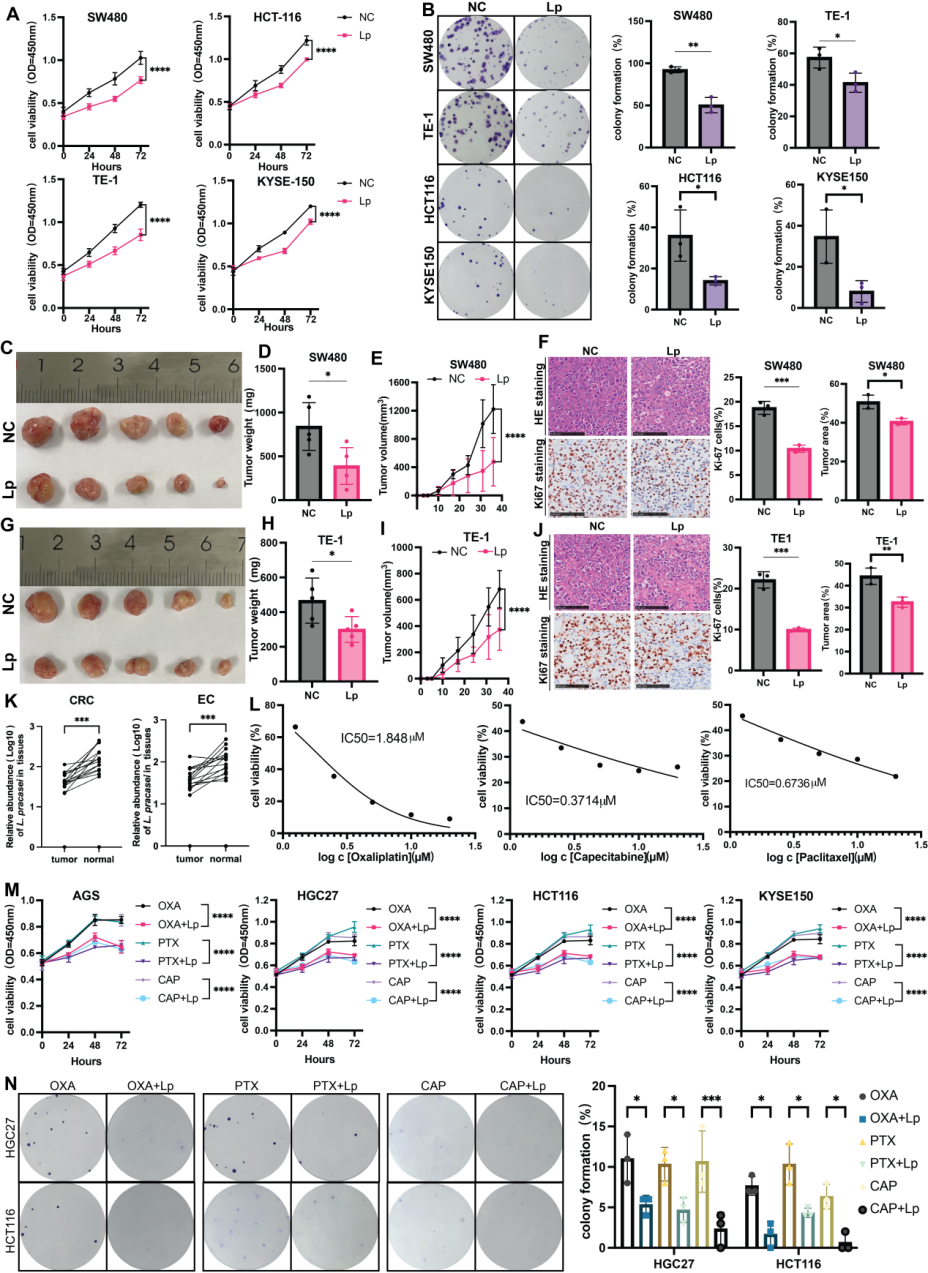
***L.* paracasei ZJUZ2-3 inhibited gastrointestinal tumor growth and promoted chemotherapy responsiveness in gastrointestinal cancer.** (A) *L.paracasei* ZJUZ2-3 (MOI=100) inhibits the cell viability of gastrointestinal cancer cells SW480, HCT116, KYSE150 and TE-1 as determined by CCK8 assays. (Lp: *L. paracasei* ZJUZ2-3). (B) Colony formation of gastrointestinal cancer cells under the treatment of* L.paracasei* ZJUZ2-3. (C) The representative tumor morphologies. (D) Tumor weight of SW480 xenograft. Tumor weight was shown as mean ± SD. (E) Tumor growth curve of mice from mice injected PBS and *L.paracasei* ZJUZ2-3. (F) Representative images of H&E staining of tumor tissues of SW480 xenograft injected PBS and *L.paracasei* ZJUZ2-3. Ki-67 staining showed decreased proliferation rates in *L. paracasei* injected mice compared with PBS. scale bars, 100μm. Lp, *L. paracasei.* (G) The representative tumor morphologies. (H) Tumor weight of TE-1 xenograft. (I) Tumor growth curve of mice from mice injected PBS and *L.paracasei* ZJUZ2-3. (J) Representative images of H&E staining of tumor tissues of TE-1 xenograft injected PBS and *L.paracasei* ZJUZ2-3. Ki-67 staining showed decreased proliferation rates in *L. paracasei* injected mice compared with PBS. scale bars, 100μm. Lp, *L. paracasei.* (K) The relative abundance of *L. paracasei* in tumor tissues and normal tissues of EC and CRC patients. (L) IC50 curves of Oxaliplatin, Capecitabine and Paclitaxel. (M) *L.paracasei* ZJUZ2-3 (MOI=100) enhances the inhibitory effect of chemotherapy drugs on the cell vitality of gastrointestinal cancer cells SW480, HCT116, KYSE150 and TE-1 as determined by CCK8 assays. (Lp: *L. paracasei* ZJUZ2-3). (N) Colony formation of gastrointestinal cancer cells under the treatment of chemotherapy drugs with or without *L.paracasei* ZJUZ2-3. Data in A, E, I, M and N were analyzed by two-way ANOVA, Data in B, D, H, J and K were analyzed by t tests; Means ± SD. ns ≧0.05, *p < 0.05, **p < 0.01, ***p < 0.001, ****p < 0.0001.

Our findings in gastric cancer demonstrate significant tumor-suppressive effects of *L. paracasei* ZJUZ2-3. To investigate whether this probiotic strain exhibits similar activity in other gastrointestinal tumors, we performed qPCR analysis on matched tumor and adjacent normal tissues from 30 esophageal cancer and 30 colorectal cancer patients. Consistent with our gastric cancer observations, *L. paracasei* was detectable in both tumor and normal tissues of half of patients (Figure 8K), with significantly higher abundance in normal tissues compared to paired tumor samples (*p*<0.001, paired t-test). These results suggest a potential conserved, tumor-suppressive role of *L. paracasei* across multiple gastrointestinal cancers.

Consistent with our hypothesis, *Lactobacillus paracasei* ZJUZ2-3 exhibited significant anti-tumor activity, demonstrating inhibition of both cell viability (p<0.0001, two-way ANOVA, CCK-8 assay) and proliferative capacity (p<0.01, t tests, colony formation assay) in human esophageal (KYSE-150 and TE-1) and colorectal (SW480 and HCT116) cancer cell lines (Figure 8A, B). We then tested whether our newly isolated *L. paracasei* ZJUZ2-3 suppresses tumor growth at gastrointestinal cancer level. We evaluated the effect of *L. paracasei* ZJUZ2-3 in esophagus and colorectal cancer mice model. Mice were sacrificed and significant reduction in tumour weight and tumour volume was observed in the subcutaneous tumor (Figure 8C, D, E, D, H and I) of *L. paracasei* ZJUZ2-3-treated mice, compared with control mice. Histological assessment showed that *L.* paracasei ZJUZ2-3-treated mice had a lower proportion of tumor area as compared with the control groups (Figure 8F and J). *L. paracasei* ZJUZ2-3 supplementation also markedly reduced the amount of Ki-67 positive proliferating cells (Figure 8F and J) in tumor tissues of intratumoral injection mice. As expected, *L. paracasei* ZJUZ2-3 suppresses tumor growth in gastrointestinal cancer.

Given the poor response of gastrointestinal tract tumors to current chemotherapy regimens, we investigated the potential of *L. paracasei* ZJUZ2-3 as a chemotherapeutic adjuvant. We systematically evaluated the median inhibitory concentration (IC50) of HGC27 by three commonly used clinical chemotherapy drugs using the standard CCK-8 method, including: oxaliplatin (1.848±0.8 μM), capecitabine (0.3714±0.021 μM), and paclitaxel (0.6736±0.035 μM). Five concentration gradients (0.1×-10× clinical blood drug concentration) were set up in the experiment, and three duplicates were set for each concentration (Figure 8L). Using CCK-8 assays (Figure 8M) and colony formation assay (Figure 8N), we demonstrated that *L.paracasei* ZJUZ2-3 significantly enhances the cytotoxic effects of standard chemotherapeutic drugs (oxaliplatin, paclitaxel, and capecitabine) against gastrointestinal cancer cells (*p*<0.0001 vs chemotherapy alone). These findings position *L.paracasei* ZJUZ2-3 as a promising microbiome-based adjunct to improve therapeutic outcomes in digestive tract malignancies.

## Discussion

In this study, we investigated the protective role of *L. paracasei* against gastric tumorigenesis. We confirmed the depletion of *L. paracasei* in tumor tissues with our in-house cohort, which was consistent with our published results based on16s rRNA gene sequencing 8, and led to uncovering a suppressive role of *L. paracasei* against GC.

By using FISH, qPCR and PCR, we confirmed the existence and enrichment of *L. paracasei* in normal tissues of GI cancer patients and a strain (ZJUZ2-3) of *L. paracasei* was successfully isolated. We further showed that the tissue-borne *L. paracasei* significantly reduced tumor weight and size in nude mice. Moreover, GC cells viability was suppressed by this bacterium. Previous studies have revealed that some *Lactobacillus* spp., such as *L. acidophilus* and *L. plantarum* could inhibit *H. pylori* colonization and gastric inflammation 25 and that *Lactobacillus*-derived short chain fatty acids (SCFA) could act as a signal for modulating colonic epithelium 26. However, this is the first study to characterize *L. paracasei* as a gastric tumor-suppressive probiotic *in vivo* and i*n vitro*.

Probiotics produce different kinds of molecules including proteins and metabolites to confer health benefits 27,28. Our results further indicated that the tumor-suppressing effect of *L. paracasei* ZJUZ2-3 is attributed to its non-protein metabolites.

A key finding of this study is that the* L. paracasei* ZJUZ2-3-derived IAA inhibited gastric tumor formation. *Lactobacillus* expresses tryptophase, which converts tryptophan into indole derivatives, such as IAA, indole-3-acetaldehyde (I3A), indole-3-lactic acid (ILA) and indole-3-propionic acid (IPA) 29. However, there has always been debate about the positive and negative association between indole metabolites and tumors. It has been reported that ILA improves colorectal tumors by regulating the epigenetic mechanism of anti-tumor infiltration of CD8+T cells 30, while supplementing with I3P can restore the growth of liver cancer cells lacking Trp, indicating that I3P is an important tumor metabolite in MYC driven liver tumors 31. Here, we illustrated the anti-tumourigenic function of IAA against the growth of GC cells and subcutaneous tumor formation in nude mice. While our study demonstrates the efficacy of IAA-producing bacteria, the direct administration of IAA itself would require a thorough screening for optimal dosage, formulation, and a complete pharmacodynamic and pharmacokinetic evaluation *in vivo*, which is a limitation of the current study and a direction for future research. Taken together, these data suggested that IAA produced by *L. paracasei* ZJUZ2-3 has a protective effect against GC.

The aryl hydrocarbon receptor (AHR) is a ligand activated transcription factor that can be activated by indole, indole derivatives, and Kynurenine (Kyn). Activated AHR participates various cellular processes. For example, it can promote the release of cytokines, including IL-17, and IL-22, thereby regulating intestinal homeostasis 32. Some indole derivatives, such as indolepropionic acid (IPA), have been shown to inhibit the release of inflammatory cytokines (IL-6, IL-1β, and TNF-α) through the activation of the aryl hydrocarbon receptor (AHR), thereby suppressing oxidative stress responses 33. Furthermore, these compounds can protect against lipid peroxidation-induced damage to the thyroid, liver, and kidneys caused by sorafenib and lenvatinib 34. Additionally, the progression of chronic kidney disease (CKD) strongly correlates with depletion of *Lactobacillus johnsonii* and reduced serum levels of its microbial metabolite indole-3-aldehyde (IAld). Therapeutic supplementation with either *L. johnsonii* or IAld ameliorates renal pathology through suppression of the aryl hydrocarbon receptor (AHR) signaling pathway 35. In hepatocellular carcinoma (HCC), AHR exerts its oncogenic effects primarily through nuclear translocation and subsequent transcriptional activation of its prototypical target gene CYP1A1, which mediates metabolic activation of procarcinogens and induces DNA damage, thereby driving tumor initiation and progression 36,37. As an indole derivative, IAA, exhibits the ability to activate AHR and suppress the NF-κB signaling pathway. We found that both *L. paracasei* ZJUZ2-3 and IAA upregulated the expression of AHR. Remarkably, both AHR antagonists CH223191 and siAHR blocked the antitumor effect, suggesting AHR is an essential molecular involving in this process. Furthermore, employing a conditional AHR knockout mouse model would provide even more robust genetic evidence to unequivocally confirm the role of the AHR pathway in the observed effects, and that this represents a logical next step in our research pipeline. Previous studies have shown that the activation of AHR could inhibit the NF-κB signaling pathway. However, the interaction between AHR and NF-κB is still unclear. We identified that the oncoprotein MTDH could interact with AHR and NF-κB by COIP-MS. Consistently, MTDH promoting hepatocyte NF-kB activity has been reported in hepatocellular carcinoma (HCC) 22. In our study, we discovered that the AHR activated by IAA could bind MTDH to reduce its phosphorylation, which subsequently inhibit the NF-κB signaling pathway. In contrast, the treatment of siAHR and CH223191 rescued the phosphorylation of MTDH and IKB, promoting NF-κB activity in GC cells. Finally, we also identified putative binding sites between AHR and MTDH using computer simulation methods. Taken together, AHR restricts GC cells NF-κB activation by interfering the interaction between MTDH and IKB.

Notably, AHR exhibits significant situational dependence in the regulation of NF-κB. While the IAA-AHR axis demonstrates pro-inflammatory effects (activating p38MAPK/NF-κB) in kidney diseases 38, this study reveals that it exerts anti-inflammatory effects in gastric cancer (inhibiting NF-κB via MTDH). This bidirectional regulation may be attributed to tissue-specific co-factors, microenvironmental metabolic stress, or differences in Hydrocarbon Receptor Nuclear Translocator (ARNT).

### Limitations

While this research provides mechanistic insights using cell lines, xenograft models. This study still has limitations. This research lacks of humanized immune system models to fully recapitulate tumor-immune interactions (planned collaboration for humanized PDX studies) and unexplored strain-specific effects across *Lactobacillus paracasei* subspecies (ongoing isolation and comparative testing of clinical isolates). These refinements will strengthen translational relevance and mechanistic depth.

## Conclusion

Using a tissue-derived isolate, we have shown that *L. paracasei* inhibits gastric tumorigenesis via its metabolite indole-3-acetic acid (IAA). IAA activates the aryl hydrocarbon receptor (AHR), which subsequently blocks the NF-κB signaling pathway by competitively binding to MTDH in tumor cells. Our results suggest that the reduction of *L. paracasei* in tumor tissues contributes to gastrointestinal cancer development, implying that supplementation with this probiotic could be a promising therapeutic approach.

The translational potential of our study is significant for several reasons: 1. *L. paracasei* is one of the most commonly used probiotics and naturally occurs in human tissues. 2. We utilized a strain of *L. paracasei* isolated from human sources. 3. We uncovered a potential role for IAA in suppressing tumor growth in patients with gastrointestinal cancers.

This study establishes a mechanistic foundation for developing novel dietary-probiotic therapies targeting microbial AHR ligands (e.g., IAA) against gastrointestinal cancers, opening clinical translation avenues. Our findings further underscore the critical role of microbiota-host crosstalk in oncotherapy, providing a robust framework for exploiting microbial metabolites in precision cancer interventions.

## Materials and Methods

### Human subjects

Tumor tissues and matched normal tissues from 90 gastric cancer patients, 30 esophageal cancer patients and 30 colorectal cancer patients receiving surgery during 2018-2020 were postoperatively collected at the First Affiliated Hospital of Zhejiang University School of Medicine.

### Bacterial isolation

The pathological specimen was obtained from a fresh normal gastric tissue specimen of a female patient who underwent radical surgery for gastric cancer at the First Affiliated Hospital of Zhejiang University School of Medicine. The specimen was collected within 2 hours after resection, and placed in PBS (Servicebio, G0002) pre-chilled at 4 ºC. The specimen was transported in an ice box and processed within 4 hours.

The tissue was washed twice with sterilized PBS, mechanically disaggregated, resuspended and diluted with TBS (Biotech, A510025). The dilutions were spread on MRS or Lactobacillus Culture Agar (Haibo Biotech, HB0384, HB0392), which were anaerobically incubated at 37 ºC for 24-48 hours. Single colonies were picked and re-diluted for culture of purified singly colonies. The obtained isolate was stored with LB or MRS containing 20% glycerol at -80 ºC.

### Bacterial culture condition

A new strain of* Lactobacillus paracasei* ZJUZ2-3 was isolated from normal gastric tissue of a GC patient. It was cultured anaerobically in Lactobacillus Broth Medium (LBM) (HB8637-2, Hopebiol) for 24 hours at 37 ºC. Overnight *L. paracasei* ZJUZ2-3 broth culture was centrifuged at 5000 g for 15 minutes and the supernatant was filtered through a 0.22μm filter to obtain the *L. paracasei* conditioned medium (Lp.CM). *E. coli* MG1655, which was used as control, was obtained from American Type Culture Collection (ATCC), and incubated aerobically in Luria Bertani (LB) broth (HB0128, Hopebiol) at 37 ºC.

### Whole genome sequencing (WGS)

The Whole Genome Sequencing (WGS) was completed by Sangon biotech (Shanghai, China). They used Illumina HiSeq and PacBio RS II sequencing system to analysis the isolated bacteria *L.paracasei* ZJUZ2-3 and obtain the whole genome.

### Cell culture

AGS (ATCC) and HGC27 (Cell Bank of the Shanghai Institute of Cell Biology, Chinese Academy of Sciences) were cultured in royal park memorial institute (RPMI) medium (Hyclone) supplemented with 10% fetal bovine serum (FBS, Gibco) and maintained at 37 ºC with 5% CO_2_.

### Cell viability assay

Cell viability was assessed using the Cell Counting Kit-8 (K1018, APExBIO). For each well in a 96-well plate, 2000 cells were seeded and treated with either bacteria-conditioned medium or direct bacterial exposure. Gastric cancer (GC) cells were exposed to the bacterial conditioned medium for four consecutive days at a concentration of 1% (vol/vol). Following this treatment, 10 μL of CCK8 solution was added to each well, and absorbance at 450 nm was measured daily after incubation at 37 ºC for two hours.

Subsequently, a colony formation assay was conducted. Five hundred cells were plated in a 6-well plate, with the treatment medium changed every two days. After culturing for 10-14 days, cells were fixed using 4% paraformaldehyde and stained with a solution of crystal violet (0.2%, catalog number: 22172503; Biosharp). Colonies containing over fifty cells were counted. All experiments were performed in triplicate across three independent trials.

### Enzyme-linked immunosorbent assay (ELISA)

The levels of indole-3-acetic acid (IAA) and aryl hydrocarbon receptor (AHR) in treated cells as well as their culture supernatants and tumor tissues from nude mice were quantified using sandwich ELISA kits according to the manufacturer's instructions (ARD13763; AoRuiDa Biology, China; YX-E11299; Yuanxin, China).

### Quantitative real-time polymerase chain reaction (qPCR) and gel electrophoresis

To assess the relative abundance of *L. paracasei* in clinical tissues and nude mice, quantitative real-time PCR (qPCR) was employed. The primer sequences utilized for detecting *L. paracasei* and total bacterial DNA were based on previously published methods 39. Specifically, for *L. paracasei*, the forward primer was 5'-CAACCGTGATGACACTG-3', and the reverse primer was 5'-CCAACGTTAATCCGGTACTG-3'. To quantify total bacteria, the 16S rRNA gene was targeted using primers described elsewhere 40: 16s rRNA, forward 5'- GGTGAATACGTTCCCGG-3', reverse 5'-TACGGCTACCTTGTTACGACTT-3'. The qPCR reactions were conducted on a StepOnePlus instrument (ABI Corp) under the following conditions: an initial denaturation at 95 ºC for 30 seconds, followed by 40 cycles of 95 ºC for 5 seconds (annealing), and 60 ºC for 30 seconds (extension). Each DNA sample was analyzed in triplicate to obtain the average cycle threshold (Ct) value. Samples with Ct value variations exceeding 2 (Ctmax - Ctmin) or undetermined Ct values were excluded from further analysis. The relative abundance of L. paracasei was calculated relative to the total bacterial load using the 2^(-ΔCt) method, where ΔCt represents the difference between the mean Ct value of L. paracasei and the mean Ct value of total bacteria.

Following amplification, PCR products were subjected to electrophoresis at 100V for 30 minutes, and the resulting gel images were captured using an Invitrogen iBright system (BIO-RAD, California, USA).

### SiRNA transfection

For siRNA knockdown experiments, polyplus transfection kit was used to transfect AGS, HGC27 with siRNAs targeting AHR according to the manufacturer' s protocol.

### DNA extraction

Genomic DNA was extracted from tissue samples using the QIAamp DNA Mini Kit (Qiagen, Hilden, Germany) following the manufacturer's protocol. The concentration of the isolated DNA was quantified with a Nanodrop 2000 spectrophotometer (Thermo Scientific, Waltham, MA). All extracted DNA samples were stored at -80 ºC until further PCR amplification.

### Immunoprecipitations mass spectrometry (IP-MS)

Proteins were extracted by RIPA lysis buffer containing phosphatase and protease inhibitor, which was then incubated at 4 °C overnight with protein A/G-agarose beads (YJ003, EpiZyme) and antibody together. For IP of AHR, anti-AHR antibody (Proteintech, Wuhan, China; Cat# 28727-1-AP) was used while rabbit IgG served as negative control. The beads were washed and boiled with 40 ml loading buffer. Boiled samples were subjected to SDS-PAGE and immunoblotted with anti-AHR antibody (Proteintech, Wuhan, China; Cat# 67785-1-Ig) and anti-MTDH antibody (Proteintech, Wuhan, China; Cat# 13860-1-AP). And proteins in-gel were digested for HPLC tandem mass spectrometry (LC/MS) by the First Affiliated Hospital of Zhejiang University, and finally visualized with Coomassie brilliant blue staining. The resulting MS/MS raw data were processed using Proteome Discoverer software v2.5, developed by Thermo Scientific. Tandem mass spectra were searched against SwissProt Human database using the SEQUEST algorithm.

### RNA sequencing (RNA-seq) and analysis

Total RNA was extracted from HGC27 cells treated with IAA and PBS control using Trizol reagent. RNA sequencing (RNA-seq) was conducted by Oebiotech (Shanghai, China). Gene expression levels were quantified as Fragments per Kilobase of transcript per Million mapped reads (FPKM) utilizing the DESeq2 software.

### Western blot analysis

Total protein extraction was carried out using RIPA lysis buffer supplemented with phosphatase and protease inhibitors. Protein concentrations were determined via the BCA assay (A55860; Thermo Scientific), followed by separation on a 10% SDS-PAGE gel and transfer onto PVDF membranes (Beyotime Institute of Biotechnology, Shanghai, China). The membranes were blocked with 5% BSA for one hour before being incubated overnight with rabbit antibodies against GAPDH (1:2000; catalog number: 60004-1-Ig; Proteintech), NF-κB (1:1000; catalog number: 10745-1-AP; Proteintech), IκB-α (1:1000; catalog number: ab32518; Abcam), pIκB-α (1:1000; catalog number: ab92700; Abcam), pMTDH (1:1000; PK7863; Pyram), along with mouse antibodies against AHR (1:1000; catalog number: 67785-1-Ig; Proteintech), PCNA (1:2000; #2586; Cell Signaling Techenology) and MTDH(1: 5000; 68592-1-Ig; Proteintech). Subsequently, HRP-conjugated secondary antibodies were treated, and the signal was measured using the Enhanced ECL Chemiluminescent Substrate Kit (36222ES76, Hangzhou, China). The intensity was analyzed by Image J software.

### *In vivo* xenograft model

Male nude mice (4-5 weeks, BALB/C) were obtained from Hangzhou Ziyuan Laboratory Animal Technology Company. All animal experiments were approved by the Institutional Animal Care and Use Committee at the First Affiliated Hospital of Zhejiang University. A total of 3×10^6 HGC27 cells were subcutaneously injected into the right flank of the mice. Seven days post-tumor injection, mice in the treatment group received 1×10^7 CFU Lp resuspended in 100 µL of PBS, along with either 100 µL of 10 μM IAA, or 50 μM CH223191, or a combination of CH223191+IAA via intratumoral injection once every two to three days; meanwhile, the control group was administered an equivalent volume of PBS. Mice were sacrificed two to three weeks after treatment. Tumor volume was measured bi-daily and tumor weight was recorded at study conclusion to assess the impact of Lp on tumor growth.

The establishment groups for orthotopic models of gastric cancer in mice followed similar protocols as described above. Anesthesia was induced using intraperitoneal injection of 1% pentobarbital. Following a midline abdominal incision, tumor cells were injected directly into the stomach. Seven days after tumor cell implantation, treatment groups received either 1×10^7 CFU Lp resuspended in 100 µL PBS or one hundred microliters each of either 10 μM IAA or 50 μM CH223191—or their combination—administered orally once every two to three days; control animals received an equal volume of PBS.

### Fluorescence in situ hybridization (FISH)

The Texas Red-Lab158 probe (sequence: 5'-GGTATTAGCACCTGTTTCCA-3') was employed to detect colonization of *L. paracasei* in paraffin-embedded gastric sections from mice and patients with gastric cancer (GC). Following deparaffinization and rehydration, the specimens were treated sequentially with 0.2 N HCl and Proteinase K for 10 minutes each. After incubation with a blocking buffer at 55 °C for 2 hours, the Lab158 probe (diluted 1:50 in a pre-heated hybridization buffer at 88 °C for 3 minutes) was introduced and allowed to hybridize overnight in a dark, humid chamber at 42 °C. Subsequently, the specimens were washed using a wash solution composed of 20 mM Tris-HCl (pH=7.2) and 40 mM NaCl, followed by mounting with DAPI-antifade solution (Thermo Fisher Scientific, Cat# P36931, Waltham, MA, USA). Images were captured using a fluorescent microscope (Leica).

### Hematoxylin-eosin straining

Paraffin-embedded tissue sections were initially deparaffinized and rehydrated. The slides were subsequently incubated with hematoxylin for 15 minutes, followed by an ammonia solution (0.08% in water for 10 seconds), and eosin for 30 seconds, with washing using tap water between each step. After dehydration, the slides were mounted onto cover slips using mounting media. Metastatic areas and foci were then quantified employing ImageJ software.

### Immunohistochemistry (IHC)

Immunohistochemistry (IHC) for Ki-67 antibody (Cell Signaling Technology, Cat# 12202, Danvers, MA, USA) was performed on paraffin-embedded sections measuring 4 µm in thickness. The proliferation index was calculated based on the percentage of positive cells observed. A minimum of three random fields from corpus to antrum sites were counted for each mouse at a magnification of 20X.

### Immunofluorescence (IF) staining

Anti-AHR (Proteintech, Wuhan, China; Cat# 67785-1-Ig, 1:500), anti-MTDH (Proteintech, Wuhan, China; Cat# 13860-1-AP,1:500) IF staining were performed on GC cells in confocal dishes. Images were acquired with a fluorescent microscope (Leica).

### Molecular docking analysis

3D structures of AHR and MTDH were downloaded from the PDB protein database 41, And the structure of the AHR-MTDH complex was modelled by HDOCK 42, and visualized with ChimeraX.

### Statistical analysis

Data are presented as mean ± SEM. The differences between two groups were analyzed using t-tests while comparisons among multiple groups employed Two-way ANOVA analysis to evaluate variations in cell viability. Statistical significance is indicated as follows: *p<0.05; **p<0.01; ***p<0.001; ****p<0.0001. All statistical analyses were performed using GraphPad Prism version 9 (GraphPad Software, San Diego, CA).

## Supplementary Material

Supplementary figures and table.

## Abbreviations

AHR: aryl hydrocarbon receptor
*L. paracasei*/Lp: *Lactobacillus paracasei*
GC: gastric cancer
CRC: colorectal cancer
EC: esophagus cancer
IAA: Indole-3-acetic acid
NF-kB: nuclear factor kB
*H. pylori*: *Helicobacter pylori*
SCFAs: short-chain fatty acids
Trp: tryptophan
TrpA: tryptophan synthase subunit alpha
FISH: fluorescence in situ hybridization
WGS: whole genome sequencing
CM: culture medium
Lp.CM: *L.paracasei* conditional medium
PK: protease K
PI3K: phosphatidylinositol 3-kinase
GSEA: Gene set enrichment analysis
DEGs: differential expressed genes
Co-IP: Co-immunoprecipitation
LC-MS/MS: liquid chromatography and high-throughput mass spectrometry
MTDH: Metadherin
TCGA: The Cancer Genome Atlas
TNF-α: tumor necrosis factor α
I3A: indole-3-acetaldehyde
ILA: indole-3-lactic acid
IPA: indole-3-propionic acid
Kyn: Kynurenine
HCC: hepatocellular carcinoma
LBM: Lactobacillus Broth Medium
ATCC: American Type Culture Collection
RPMI: royal park memorial institute
FBS: fetal bovine serum
CCK8: Cell Counting Kit-8
ELISA: Enzyme-linked immunosorbent assay
qPCR: Quantitative Real-Time Polymerase Chain Reaction
IP-MS: Immunoprecipitations mass spectrometry
RNA-seq: RNA sequencing
HE: Hematoxylin-eosin straining
IHC: Immunohistochemistry
IF: Immunofluorescence
