# Supplementary material for: Lactobacillus paracasei ZJUZ2-3 inhibits gastrointestinal tumors via the IAA-induced AHR/MTDH/NF-κB axis
Source: Int J Biol Sci. 2025 Oct 10;21(14):6522–41. doi: 10.7150/ijbs.114602 (PMC12594602; doi:10.7150/ijbs.114602)
Supplement: Supplementary file 1 — Supplementary figures and table. [file ijbsv21p6522s1.pdf]

Supplementary information

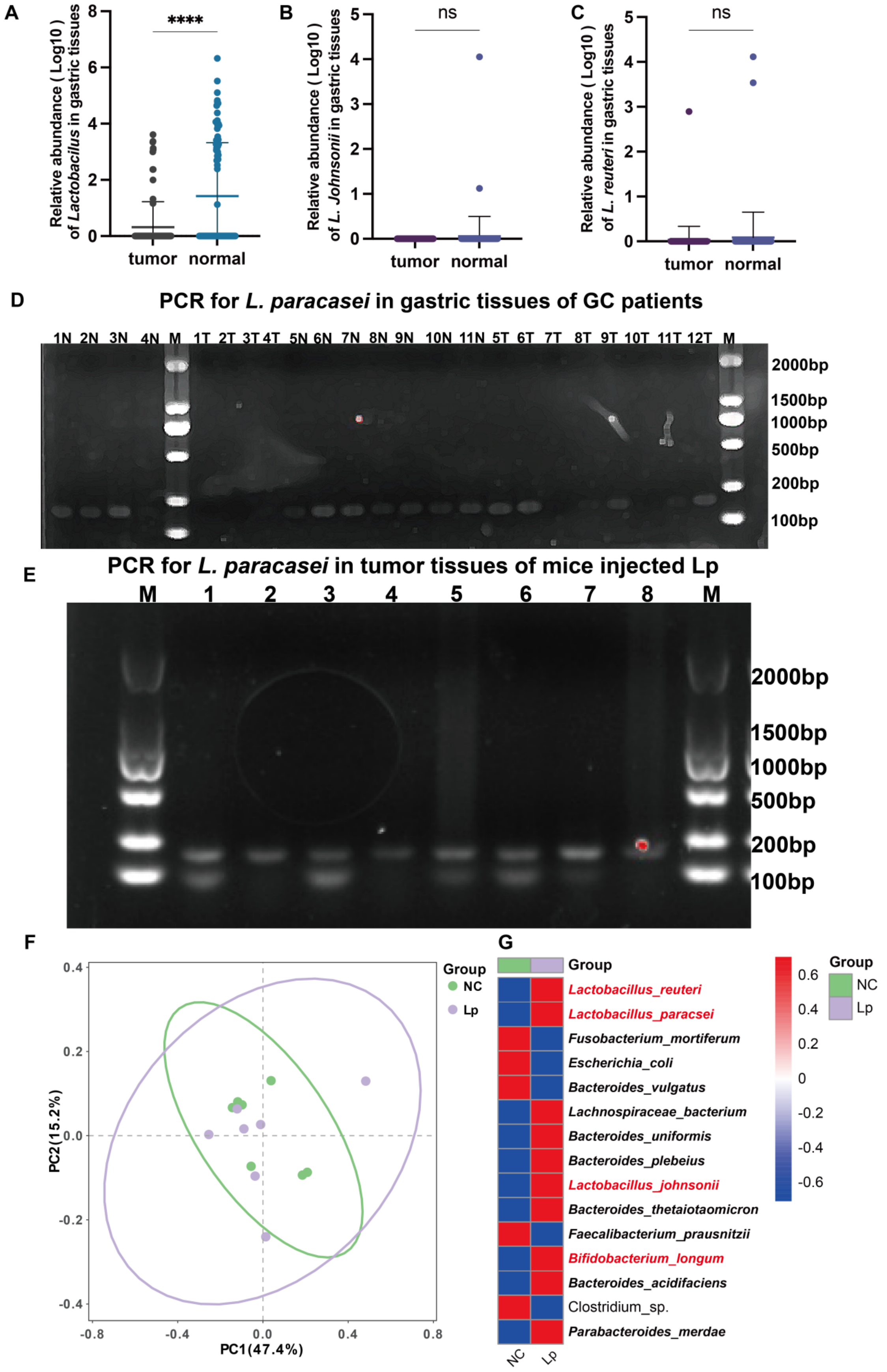

Supplementary Figure 1

(A-C) The relative abundance of (A) *Lactobacillus* (B) *L. johnsonii* (C) *L. reuteri* in tumor tissues and normal tissues of GC patients. Data were analyzed by t tests; Means ± SD. ns $p \geqq 0.05$, **p* < 0.05, ** *p* < 0.01, ****p* < 0.001, **** *p* < 0.0001.

(D) PCR was used to determine the colonization of *L. paracasei* in both tumor or normal tissues of GC patients.

(E) PCR was used to determine the colonization of *L. paracasei* in tumor tissues of HGC27 xenograft.

(F) Beta-diversity by PCoA based on Binary-jaccard dissimilarity matrix in stomach samples of nude mice.

(G) Heatmap of differential bacterial species (p<0.05) in nude mice, compared with control groups.

PCoA, principal coordinate analysis.

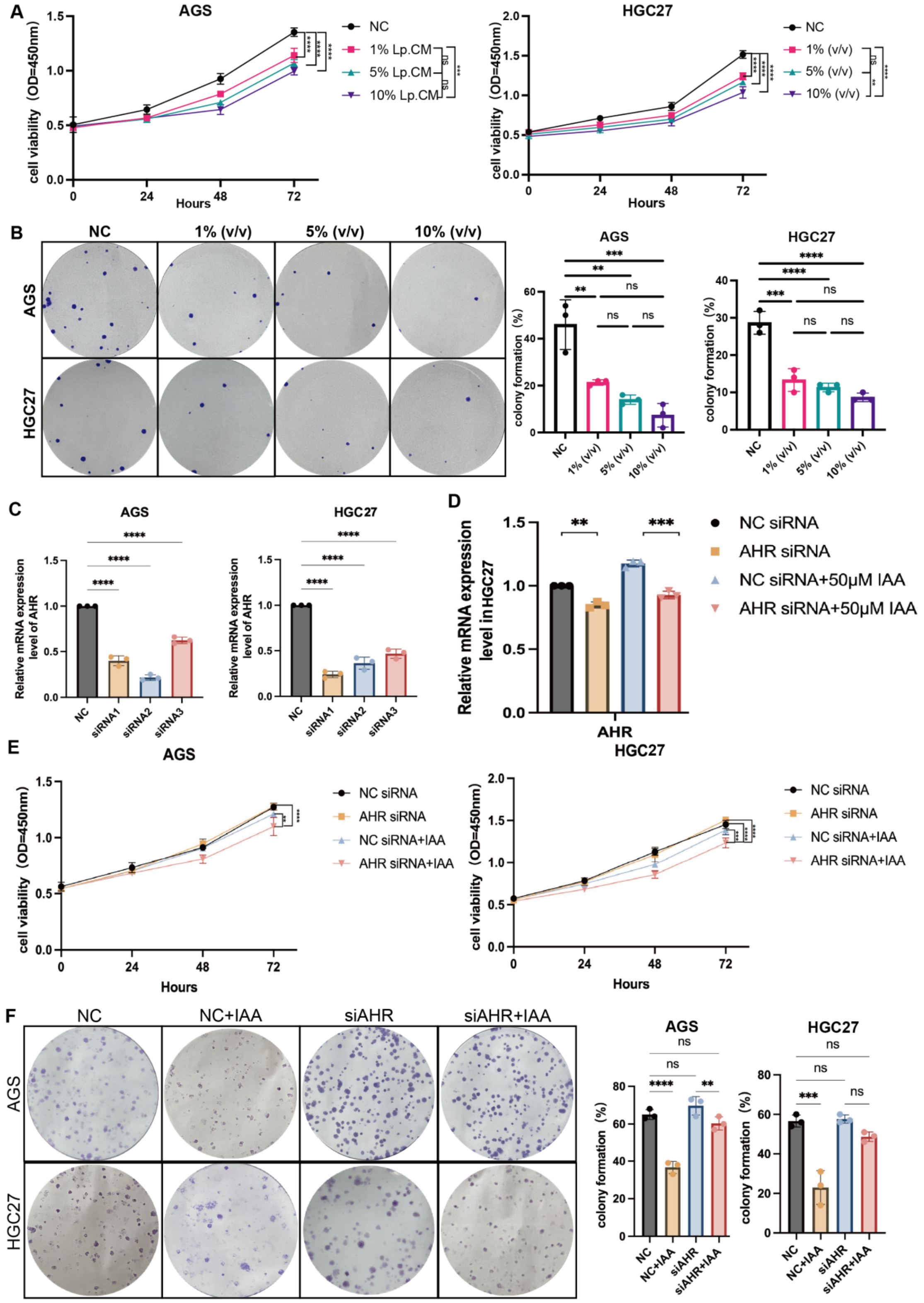

Supplementary Figure 2

(A, B) (A)Cell viability at OD450nm (B) colony formation of human GC cells with PBS, 1% Lp.CM, 5% Lp.CM and 10% Lp.CM.

(C) The knockdown effect of AHR of human GC cells through qPCR.

(D) The mRNA level of AHR of HGC27 with siAHR and IAA.

(E, F) (E)Cell viability at OD450nm (F) colony formation of human GC cells with PBS, IAA, siAHR and siAHR+IAA. Data were analyzed by two-way ANOVA; Means ± SD.

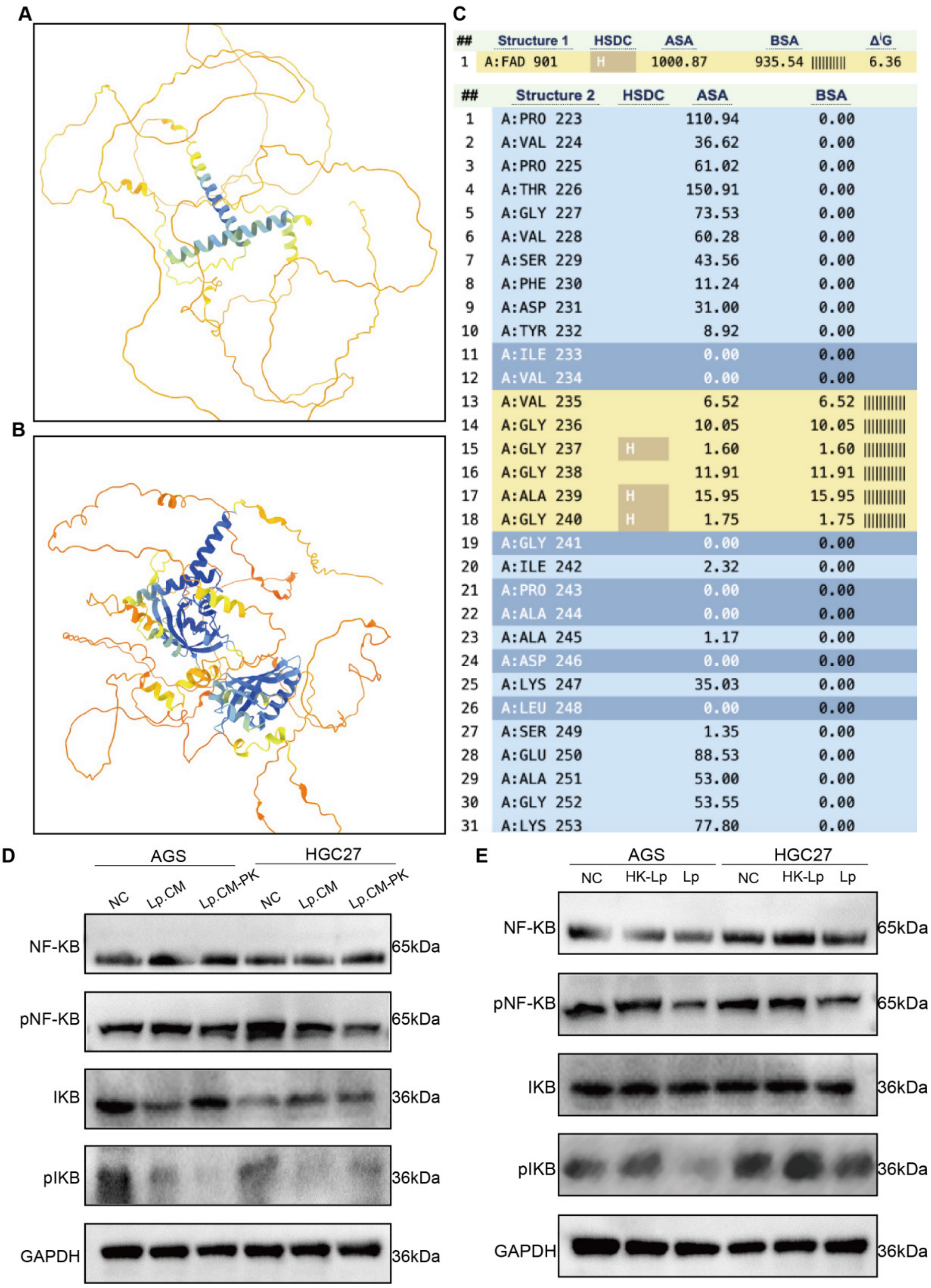

Supplementary Figure 3

(A, B) The 3D structure of (A)MTDH and (B)AHR using ChimeraX.

(A) The receptor protein is located above, and the ligand protein is located below. Each row contains an amino acid residue, with the yellow region indicating the predicted binding region. HSDC is predicted for hydrogen bonding, BSA is the area of interaction encapsulation, and the more vertical lines there are, the higher the degree of encapsulation, indicating a tighter interaction.

(D) Western blot analysis of the inhibition of NF-kB signaling pathway of AGS and HGC27 treated with Lp. CM and Lp. CM-PK.

Table 1 The proteins binding to AHR by COIP

Checked	Protein FD	Master	Accession	Descriptio	Exp. q-val	Sum PEP	Coverage	# Peptides	# PSMs	# Unique I	# AAs	MW [kI	calc. pI	Score Seq	# Peptides	Biological	Cellular Cc	Molecular	Pfam IDs	Entrez Ge	Ensembl (	Gene Sym	Gene ID	WikiPathw	Reactome	# Protein I	Found in !	# Protein (
TRUE	High	Master Pr	CON_P13	SWISS-PF	0	56.701	19	15	83	12	593	59.5	5.21	148.42	15											0	High	1
TRUE	High	Master Pr	P04264	Keratin, ty	0	44.581	19	11	37	10	644	66	8.12	48.25	11	cell organi	non-struct	bone, toot	Pf00038,	3848	ENSG000	KRT1	B2RA01; I	Corticotro	Formation	4	High	1
TRUE	High	Master Pr	P35908	Keratin, ty	0	36.324	21	14	35	11	639	65.4	8	60.47	14	cell cycle	plasma m	bone, toot	Pf00038,	3849	ENSG000	KRT2	hsa:3849;		Formation	2	High	1
TRUE	High	Master Pr	CON_P02	SWISS-PF	0	27.935	17	10	11	10	607	69.2	6.18	16.15	10	stress resp	non-struct	nucleic ac	Pf00273	280717		ALB	A5PJX3; b			0	High	1
TRUE	High	Master Pr	P02533	Keratin, ty	0	24.795	17	8	36	5	472	51.5	5.16	51.39	8	cell organi	plasma m	cytoskelet	Pf00038	3861	ENSG000	KRT14	hsa:3861;	Corticotro	Formation	4	High	1
TRUE	High	Master Pr	P16403	Histone H	0	21.438	24	5	16	5	213	21.4	10.93	31.89	5	cell organi	nucleus;ot	nucleic ac	Pf00538	3006	ENSG000	H1-2	A8K4I2; hs		Apoptosis	2	High	1
TRUE	High	Master Pr	CON_P35	SWISS-PF	0	20.05	13	8	14	8	623	62.1	5.3	11.88	8											0	High	1
TRUE	High	Master Pr	Q7Z794	Keratin, ty	0	17.572	10	6	10	5	578	61.9	5.99	11.54	6	cell organi	plasma m	bone, toot	Pf00038,	374454	ENSG000	KRT77	hsa:37445		Formation	2	High	1
TRUE	High	Master Pr	P13647	Keratin, ty	0	16.636	11	6	10	4	590	62.3	7.74	21.76	6	cell organi	cytosol;cy	bone, toot	Pf00038,	3852	ENSG000	KRT5	hsa:3852;		Formation	3	High	1
TRUE	High	Master Pr	P16401	Histone H	0	14.931	11	3	10	3	226	22.6	10.92	26.73	3	cell organi	nucleus;ot	nucleic ac	Pf00538	3009	ENSG000	H1-5	hsa:3009;		Apoptosis	2	High	1
TRUE	High	Master Pr	P49756	RNA-bindi	0	13.595	6	4	4	4	843	100.1	6.32	5.59	4	RNA meta	nucleus;ot	nucleic ac	Pf00076,	58517	ENSG000	RBM25	A0PJL9; E		mRNA Spl	1	High	1
TRUE	High	Master Pr	P11387	DNA topoi	0	11.542	6	6	9	6	765	90.7	9.31	9.31	6	cell cycle	nucleus;ot	nucleic ac	Pf01028,	7150	ENSG000	TOP1	A8KA78; E	Ebola viru	SUMOylat	3	High	1
TRUE	High	Master Pr	Q8NE71	ATP-bindi	0	9.417	4	3	4	3	845	95.9	6.8	4.31	3	protein me	cytosol;tra	translation	Pf00005	23	ENSG000	ABCF1	A2BF75; I	IL-18 sign	ABC-famil	2	High	1
TRUE	High	Master Pr	Q9GZR7	ATP-depe	0	8.863	5	4	5	4	859	96.3	9.06	6.29	4	RNA meta	nucleus	other mole	Pf00270,	57062	ENSG000	DDX24	E7EMJ4; I			0	High	1
TRUE	High	Master Pr	Q9UQ88	Cyclin-dep	0	8.835	4	3	4	3	783	91.3	5.36	4.27	3	cell cycle	nucleus;ot	kinase act	Pf00069	728642	ENSG000	CDK11A	hsa:72864	1p36 copy	Recruitme	2	High	1
TRUE	High	Master Pr	Q00839	Heterogen	0	8.673	5	4	7	4	825	90.5	6	4.28	4	cell cycle	cytosol;cy	cytoskelet	Pf00622,	3192	ENSG000	HNRNPU	hsa:3192;	mRNA prc	mRNA Spl	4	High	1
TRUE	High	Master Pr	Q9NR30	Nucleolar	0	8.537	6	4	5	4	783	87.3	9.28	4.24	4	cell organi	cytosol;mi	enzyme re	Pf00270,	9188	ENSG000	DDX21	B2RDL0; I	10q11.21c	Major path	3	High	1
TRUE	High	Master Pr	Q9H0A0	RNA cytid	0	8.402	4	4	8	4	1025	115.7	8.27	3.4	4	protein me	nucleus;ot	nucleic ac	Pf05127,	55226	ENSG000	NAT10	B4DFD5; I	Metapathw	rRNA moc	2	High	1
TRUE	High	Master Pr	Q9UHX1	Poly(U)-bi	0	8.336	3	3	3	3	559	59.8	5.29	6.48	3	cell organi	nucleus;ot	nucleic ac	Pf00076	22827	ENSG000	PUF60	A8K8K8; I		mRNA Spl	1	High	1
TRUE	High	Master Pr	Q07020	60S riboso	0	7.426	19	3	4	3	188	21.6	11.72	3.39	3	protein me	cytosol;EF	nucleic ac	Pf17135	6141	ENSG000	RPL18	F8VWC5;	Cytoplasm	Major path	14	High	1
TRUE	High	Master Pr	CON_P12	SWISS-PF	0	7.4	7	2	4	2	359	38.4	5.5	5.16	2	stress resp	non-struct	enzyme re	Pf00031	280988	ENSBTAG	AHSG	A6QLF7; E		Neutrophil	4	High	1
TRUE	High	Master Pr	P46087	Probable 2	0	7.257	4	3	4	3	812	89.2	9.23	4	3	cell organi	nucleus	other mole	Pf01189,	4839	ENSG000	NOP2	A1A4Z3; E	Imatinib ar	rRNA moc	3	High	1
TRUE	High	Master Pr	O76094	Signal rec	0	6.753	4	2	2	2	671	74.6	9.26	2.5	2	other biolc	cytosol;EF	nucleic ac	Pf08492,	6731	ENSG000	SRP72	G5E9Z8; I		SRP-depe	1	High	1
TRUE	High	Master Pr	O60524	Nuclear ex	0	6.223	3	3	5	3	1076	122.9	6.35	6.41	3	cell organi	cytosol;tra	translation	Pf05670,	9147	ENSG000	NEMF	A0JLQ3; E			0	High	1
TRUE	High	Master Pr	P15924	Desmopla	0	6.15	1	4	5	4	2871	331.6	6.81	0	4	cell adhes	plasma m	cytoskelet	Pf00681,	1832	ENSG000	DSP	B2RTT2; D	Arrhythmo	Formation	9	High	1
TRUE	High	Master Pr	CON_P00	Trypsin - S	0	6.024	8	2	124	2	231	24.4	7.18	103.2	2	protein me	non-struct	other mole	Pf00089							0	High	1
TRUE	High	Master Pr	Q02878	60S riboso	0	5.59	10	3	4	3	288	32.7	10.58	2.37	3	cell organi	cytosol;EF	nucleic ac	Pf01159,	6128	ENSG000	RPL6	hsa:6128;	Cytoplasm	SRP-depe	14	High	1
TRUE	High	Master Pr	P50914	60S riboso	0	5.51	11	2	3	2	215	23.4	10.93	5.39	2	protein me	cytosol;tra	other mole	Pf01929	9045	ENSG000	RPL14	hsa:9045;	Cytoplasm	SRP-depe	14	High	1
TRUE	High	Master Pr	P36578	60S riboso	0	5.454	10	4	4	4	427	47.7	11.06	0	4	protein me	cytosol;EF	other mole	Pf00573,	6124	ENSG000	RPL4	A8K502; I	Cytoplasm	SRP-depe	14	High	1
TRUE	High	Master Pr	P31944	Caspase-1	0	5.397	9	2	3	2	242	27.7	5.58	6.08	2	protein me	plasma m	other mole	Pf00656	23581	ENSG000	CASP14	hsa:23581	Vitamin D	Formation	2	High	1
TRUE	High	Master Pr	Q7RTS7	Keratin, ty	0	5.197	4	2	3	1	529	57.8	7.71	3.91	2	cell organi	cytosol;cy	bone, toot	Pf00038,	121391	ENSG000	KRT74	B5MD61;		Formation	2	High	1
TRUE	High	Master Pr	O60841	Eukaryotic	0	5.058	1	1	3	1	1220	138.7	5.49	4.59	1	cell organi	cytosol;otl	nucleic ac	Pf00009,	9669	ENSG000	EIF5B	hsa:9669;	Translatio	GTP hydro	3	High	1
TRUE	High	Master Pr	Q5SSJ5	Heterochr	0	4.933	4	2	3	2	553	61.2	9.67	1.95	2	cell organi	nucleus;ot	nucleic ac	Pf00538	50809	ENSG000	HP1BP3	A6NI71; A			0	High	1
TRUE	High	Master Pr	CON_ENS	(Bos tauru	0	4.816	6	2	3	2	401	43.9	5.35	5.29	2											0	High	1
TRUE	High	Master Pr	Q13435	Splicing fa	0	4.414	2	2	3	2	895	100.2	5.67	0	2	cell organi	nucleus		Pf02037,	10992	ENSG000	SF3B2	A8K485; E	mRNA prc	mRNA Spl	3	High	1
TRUE	High	Master Pr	Q9Y5B9	FACT com	0	4.389	2	2	2	2	1047	119.8	5.66	2.57	2	cell organi	nucleus	other mole	Pf00557,	11198	ENSG000	SUPT16H	hsa:11198		Formation	12	High	1
TRUE	High	Master Pr	O75339	Cartilage i	0	4.334	2	1	4	1	1184	132.5	8.41	8.3	1	other biolc	non-struct		Pf00090,	8483	ENSG000	CILP	B2R8F7; I		Signaling I	1	High	1
TRUE	High	Master Pr	P62805	Histone H	0	4.247	21	2	2	2	103	11.4	11.36	1.83	2	cell organi	non-struct	nucleic ac	Pf15511	121504; 5	ENSG000	H4C1; H4	A2VCL0; I	Gastric ca	RMTs met	52	High	1
TRUE	High	Master Pr	P62424	60S riboso	0	4.237	9	2	2	2	266	30	10.61	1.82	2	protein me	other mem	other mole	Pf01248	6130	ENSG000	RPL7A	hsa:6130;	Cytoplasm	Major path	14	High	1
TRUE	High	Master Pr	Q9UKV3	Apoptotic	0	4.185	2	2	2	2	1341	151.8	6.43	0	2	cell organi	plasma m	nucleic ac	Pf02037,	22985	ENSG000	ACIN1	B2RTT4; D		mRNA Spl	2	High	1
TRUE	High	Master Pr	O76021	Ribosoma	0	4.15	5	2	2	2	490	54.9	10.13	1.92	2	RNA meta	cytosol;tra	nucleic ac	Pf00687	26156	ENSG000	RSL1D1	B4DJ58; D			0	High	1
TRUE	High	Master Pr	P62241	40S riboso	0	4.114	11	2	3	2	208	24.2	10.32	2.34	2	protein me	cytosol;EF	other mole	Pf01201	6202	ENSG000	RPS8	hsa:6202;	Cytoplasm	SRP-depe	19	High	1
TRUE	High	Master Pr	P47914	60S riboso	0	4.03	9	1	3	1	159	17.7	11.66	7.09	1	protein me	cytosol;tra	other mole	Pf01779	6159	ENSG000	RPL29	A8K0H3; E	Cytoplasm	Regulation	14	High	1
TRUE	High	Master Pr	Q96GQ7	Probable A	0	3.824	4	3	3	3	796	89.8	9.28	0	3	RNA meta	nucleus;ot	other mole	Pf00270,	55661		DDX27	A0AVB6; E			0	High	1
TRUE	High	Master Pr	P23246	Splicing fa	0	3.737	4	2	2	2	707	76.1	9.44	2.12	2	cell organi	cytosol;nu	nucleic ac	Pf00076,	6421	ENSG000	SFPQ	hsa:6421;	mRNA prc	Suppressi	4	High	1
TRUE	High	Master Pr	Q6UWP8	Suprabasi	0	3.324	3	1	2	1	590	60.5	7.01	2.01	1				Pf21009	374897	ENSG000	SBSN	A8K5J0; E			0	High	1
TRUE	High	Master Pr	P49848	Transcript	0	3.296	2	1	1	1	677	72.6	8.6	2.15	1	cell organi	cytosol;nu	signal tran	Pf02969,	6878	ENSG000	TAF6	A4D2B2; A	Eukaryotic	RNA Polyr	11	High	1
TRUE	High	Master Pr	Q96SB4	SRSF prot	0	3.194	2	1	2	1	655	74.3	6.16	3.71	1	cell cycle	plasma m	kinase act	Pf00069	6732	ENSG000	SRPK1	B4DS61; I	VEGFA-VE	Replacem	4	High	1
TRUE	High	Master Pr	Q8IWX8	Calcium h	0	3.175	1	1	1	1	916	103.6	9.04	0	1	RNA meta	other mem	other mole	Pf01585,	10523	ENSG000	CHERP	hsa:10523		mRNA Spl	1	High	1
TRUE	High	Master Pr	Q9NVP1	ATP-depe	0	2.978	1	1	1	1	670	75.4	9.5	2.53	1	RNA meta	nucleus;ot	other mole	Pf00270,	8886	ENSG000	DDX18	hsa:8886;			0	High	1
TRUE	High	Master Pr	Q13428	Treacle pr	0	2.893	2	2	2	2	1488	152	9.04	0	2	RNA meta	cytosol;nu	other mole	Pf03546	6949	ENSG000	TCOF1	A0JLU0; E			0	High	1
TRUE	High	Master Pr	Q9NW13	RNA-bindi	0	2.728	2	1	1	1	759	85.7	9.22	0	1	RNA meta	nucleus		Pf00076	55131	ENSG000	RBM28	A4D100; E		Major path	1	High	1
TRUE	High	Master Pr	P62753	40S riboso	0	2.531	3	1	1	1	249	28.7	10.84	1.76	1	cell cycle	cytosol;EF	other mole	Pf01092	6194	ENSG000	RPS6	hsa:6194;	IL-5 signal	SRP-depe	49	High	1
TRUE	High	Master Pr	P62851	40S riboso	0	2.527	8	1	5	1	125	13.7	10.11	10.18	1	protein me	cytosol;tra	other mole	Pf03297	6230	ENSG000	RPS25	B2R4M7; I	Cytoplasm	Major path	19	High	1
TRUE	High	Master Pr	Q8WUA2	Peptidyl-p	0	2.295	2	1	1	1	492	57.2	5.92	0	1		cytosol;nu	other mole	Pf00076,	85313	ENSG000	PPIL4	B2RD34; I		mRNA Spl	1	High	1
TRUE	High	Master Pr	Q8TDD1	ATP-depe	0	2.164	1	1	1	1	881	98.5	10.02	0	1	RNA meta	ER/Golgi;r	signal tran	Pf00270,	79039	ENSG000	DDX54	hsa:79039			0	High	1
TRUE	High	Master Pr	P04406	Glyceralde	0	1.987	4	1	1	1	335	36	8.46	0	1	cell organi	plasma m	enzyme re	Pf00044,	2597	ENSG000	GAPDH	E7EUT4; I	Cori cycle	Glycolysis	16	High	1
TRUE	High	Master Pr	O14776	Transcript	0	1.969	1	1	1	1	1098	123.8	8.65	0	1	RNA meta	nucleus	other mole	Pf00397,	10915	ENSG000	TCERG1	hsa:10915		mRNA Spl	1	High	1
TRUE	High	Master Pr	P04040	Catalase C	0	1.961	2	1	1	1	527	59.7	7.39	0	1	protein me	non-struct	other mole	Pf00199,	847	ENSG000	CAT	A8K6C0; E	Selenium	Peroxisom	12	High	1
TRUE	High	Master Pr	Q12906	Interleukin	0	1.941	1	1	1	1	894	95.3	8.76	1.81	1	protein me	mitochonc	nucleic ac	Pf00035,	3609	ENSG000	ILF3	A8K6F2; C		PKR-medi	2	High	1
TRUE	High	Master Pr	Q9BVP2	Guanine n	0	1.903	2	1	1	1	549	62	9.16	0	1	other biolc	nucleus;ot	nucleic ac	Pf01926,	26354	ENSG000	GNL3	B2RDC1; I		Major path	1	High	1
TRUE	High	Master Pr	Q13823	Nucleolar	0	1.896	2	1	1	1	731	83.6	9.25	0	1	other biolc	nucleus	other mole	Pf01926,	29889	ENSG000	GNL2	hsa:29889			0	High	1
TRUE	High	Master Pr	Q99442	Translocat	0	1.892	2	1	1	1	399	45.8	7.12	0	1	transport;c	other mem	other mole	Pf03839	7095	ENSG000	SEC62	D3DNQ0;			0	High	1
TRUE	High	Master Pr	P01040	Cystatin-A	0	1.888	12	1	1	1	98	11	5.5	0	1	cell adhes	non-struct	enzyme re	Pf00031	1475	ENSG000	CSTA	hsa:1475;	8p23.1 co	Formation	2	High	1
TRUE	High	Master Pr	Q9BZE4	GTP-bindi	0	1.864	2	1	1	1	634	73.9	9.5	1.72	1	RNA meta	other mem	other mole	Pf06858,	23560	ENSG000	GTPBP4	B3KMC5;			0	High	1
TRUE	High	Master Pr	B9A064	Immunogl	0	1.862	4	1	1	1	214	23	8.84	0	1	other biolc	other cell	other mole	Pf07654	10042306	ENSG000	IGLL5	B9A064; I			0	High	1
TRUE	High	Master Pr	Q9UGP8	Translocat	0	1.842	1	1	1	1	760	87.9	5.31	0	1	other meta	other mem	transporte	Pf00226,	11231	ENSG000	SEC63	hsa:11231			0	High	1
TRUE	High	Master Pr	Q6PJE2	POM121 a	0	1.793	8	1	1	1	187	20.6	7.18	0	1		other mem		Pf00100	22932	ENSG000	POMZP3	F6STJ3; h			0	High	1
TRUE	High	Master Pr	O14744	Protein arg	0	1.785	2	1	2	1	637	72.6	6.29	0	1	cell organi	cytosol;EF	nucleic ac	Pf05185,	10419	ENSG000	PRMT5	A8MTP3;	Transcript	snRNP As	5	High	1
TRUE	High	Master Pr	P13639	Elongation	0	1.758	1	1	1	1	858	95.3	6.83	0	1	protein me	non-struct	translation	Pf00009,	1938	ENSG000	EEF2	A0A384N6	Translatio	Neutrophil	9	High	1
TRUE	High	Master Pr	Q99575	Ribonucle	0	1.719	1	1	1	1	1024	114.6	9.22	0	1	RNA meta	nucleus;ot	nucleic ac	Pf06978,	10940	ENSG000	POP1	A8K5W9;		tRNA proc	1	High	1
TRUE	High	Master Pr	P48741	Putative h	0	1.706	3	1	1	1	367	40.2	7.87	1.8	1	protein me	nucleus;ot	other mole	Pf00012			HSPA7	P19790; F		Regulation	1	High	1
TRUE	High	Master Pr	P02100	Hemoglob	0	1.69	7	1	2	1	147	16.2	8.63	0	1	other meta	cytosol;otl	other mole	Pf00042	3046	ENSG000	HBE1	hsa:3046;		Factors in	1	High	1
TRUE	High	Master Pr	O15446	DNA-direc	0	1.675	3	1	1	1	510	55	8.51	0	1	RNA meta	cytosol;mi		Pf08208	10849	ENSG000	POLR1G	hsa:10849	2q13 copy	B-WICH c	6	High	1
TRUE	High	Master Pr	P20930	Filaggrin C	0	1.611	0	1	1	1	4061	434.9	9.25	0	1	protein me	plasma m	bone, toot	Pf01023,	2312	ENSG000	FLG	hsa:2312;		Formation	1	High	1
TRUE	High	Master Pr	Q5C9Z4	Nucleolar	0	1.599	2	1	1	1	860	96.2	8.1	0	1	other biolc	nucleus		Pf02847,	64434	ENSG000	NOM1	hsa:64434			0	High	1
TRUE	High	Master Pr	Q9Y3U8	60S riboso	0	1.573	10	1	1	1	105	12.2	11.59	0	1	protein me	cytosol;tra	other mole	Pf01158	25873	ENSG000	RPL36	B2R4Y1; D	Cytoplasm	SRP-depe	14	High	1
TRUE	High	Master Pr	Q02413	Desmogle	0	1.566	1	1	1	1	1049	113.7	5.03	0	1	cell adhes	plasma m	other mole	Pf00028,	1828	ENSG000	DSG1	B7Z845; h		Formation	6	High	1
TRUE	High	Master Pr	Q9BRD0	BUD13 hc	0	1.536	2	1	1	1	619	70.5	9.86	0	1	cell organi	nucleus		Pf09736	84811	ENSG000	BUD13	A8K0S0; I		mRNA Spl	1	High	1
TRUE	High	Master Pr	Q96GA3	Protein LT	0	1.508	3	1	1	1	475	54.8	4.91	0	1	transport;c	cytosol;nu		Pf04180	84946	ENSG000	LTV1	hsa:84946		Major path	1	High	1
TRUE	High	Master Pr	O00268	Transcript	0	1.499	1	1	1	1	1085	110	9.94	0	1	cell organi	cytosol;nu	signal tran	Pf05236,	6874	ENSG000	TAF4	A6NGD9;		RNA Polyr	9	High	1
TRUE	High	Master Pr	Q969Q0	60S riboso	0	1.494	8	1	1	1	106	12.5	10.65	0	1	protein me	plasma m	other mole	Pf00935	6166	ENSG000	RPL36AL	hsa:6166;		Major path	13	High	1
TRUE	High	Master Pr	Q14684	Ribosoma	0	1.481	1	1	1	1	758	84.4	9.76	1.61	1	RNA meta	nucleus;ot	other mole	Pf05997	23076	ENSG000	RRP1B	hsa:23076			0	High	1
TRUE	High	Master Pr	Q8TD19	Serine/thr	0	1.46	1	1	1	1	979	107.1	5.74	0	1	cell cycle	cytosol;nu	enzyme re	Pf00069,	91754	ENSG000	NEK9	hsa:91754	Ciliopathie	EML4 and	4	High	1
TRUE	High	Master Pr	Q9H307	Pinin OS=	0	1.435	1	1	1	1	717	81.6	7.14	0	1	cell adhes	plasma m	nucleic ac	Pf04696,	5411	ENSG000	PNN	B4DZX8; I		mRNA Spl	1	High	1
TRUE	High	Master Pr	Q92841	Probable A	0	1.43	2	1	1	1	729	80.2	8.27	0	1	RNA meta	cytosol;nu	nucleic ac	Pf00270,	10521	ENSG000	DDX17	B1AHM0;	Novel intra	SUMOylat	2	High	1
TRUE	High	Master Pr	Q9NWH9	SAFB-like	0	1.427	1	1	1	1	1034	117.1	7.87	0	1	other biolc	nucleus	nucleic ac	Pf00076,	79811	ENSG000	SLTM	A8K5V8; E			0	High	1
TRUE	High	Master Pr	P35499	Sodium ch	0	1.406	0	1	1	1	1836	207.9	5.1	0	1	transport;c	plasma m	transporte	Pf00520,	6329	ENSG000	SCN4A	hsa:6329;		Interaction	2	High	1
TRUE	High	Master Pr	Q13885	Tubulin be	0	1.395	2	1	1	1	445	49.9	4.89	0	1	cell cycle	cytoskelet	cytoskelet	Pf00091,	7280	ENSG000	TUBB2A	hsa:7280;	Alzheimer'	Recycling	35	High	1
TRUE	High	Master Pr	P21810	Biglycan C	0	1.364	3	1	1	1	368	41.6	7.52	0	1	developm	non-struct	extracellul	Pf01462,	633	ENSG000	BGN	D3DWU3;		ECM prote	11	High	1
TRUE	High	Master Pr	P35579	Myosin-9	0	1.36	1	1	1	1	1960	226.4	5.6	0	1	cell cycle	plasma m	signal tran	Pf00063,	4627	ENSG000	MYH9	A8K6E4; I	Primary fo	Regulation	16	High	1
TRUE	High	Master Pr	P02452	Collagen a	0.005	1.342	1	1	1	1	1464	138.9	5.8	0	1											0	High	1
TRUE	High	Master Pr	P12111	Collagen a	0.005	1.327	1	1	2	1	3177	343.5	6.68	0	1	cell adhes	extracellul	enzyme re	Pf00014,	1293	ENSG000	COL6A3	A8MT30; I	miRNA tar	Collagen c	11	High	1
TRUE	High	Master Pr	Q7L014	Probable A	0.005	1.304	1	1	1	1	1031	117.3	9.29	0	1	cell organi	other mem	other mole	Pf00270,	9879	ENSG000	DDX46	hsa:9879;		mRNA Spl	1	High	1
TRUE	High	Master Pr	Q9UHB9	Signal rec	0.005	1.286	1	1	1	1	627	70.7	8.56	0	1	other biolc	cytosol;EF	nucleic ac	Pf16969	6730	ENSG000	SRP68	B3KUU5; I		SRP-depe	1	High	1
TRUE	High	Master Pr	Q4G0W2	Dual spec	0.005	1.263	6	1	1	1	176	18.3	8.28	0	1	other meta		other mole	Pf00782	285193	ENSG000	DUSP28	hsa:28519	2q37 copy		1	High	1
TRUE	High	Master Pr	Q01518	Adenylyl c	0.005	1.263	3	1	1	1	475	51.9	8.06	0	1	cell organi	non-struct	cytoskelet	Pf01213,	10487	ENSG000	CAP1	hsa:10487	Angiopoie	Neutrophil	7	High	1
TRUE	High	Master Pr	O00567	Nucleolar	0.005	1.262	1	1	1	1	594	66	9.19	0	1	RNA meta	nucleus;ot	nucleic ac	Pf01798,	10528	ENSG000	NOP56	hsa:10528		Major path	3	High	1
TRUE	High	Master Pr	Q86UE4	Protein LY	0.005	1.234	2	1	1	1	582	63.8	9.32	0	1	signal tran	plasma m	nucleic ac	Pf15686	92140	ENSG000	MTDH	hsa:92140	Gastric ca		3	High	1
TRUE	High	Master Pr	P26373	60S riboso	0.004	1.232	5	1	1	1	211	24.2	11.65	0	1	protein me	cytosol;EF	other mole	Pf01294	6137	ENSG000	RPL13	B4DLX3; I	Cytoplasm	SRP-depe	14	High	1
TRUE	High	Master Pr	P08670	Vimentin C	0.004	1.206	2	1	1	1	466	53.6	5.12	0	1	cell organi	plasma m	cytoskelet	Pf00038,	7431	ENSG000	VIM	B0YJC2; D	Allograft re	Aggrephag	21	High	1
TRUE	High	Master Pr	O60832	H/ACA rib	0.004	1.205	2	1	1	1	514	57.6	9.42	0	1	cell organi	nucleus;ot	nucleic ac	Pf01472,	1736	ENSG000	DKC1	F5BSB3; I		rRNA moc	2	High	1
TRUE	High	Master Pr	P46776	60S riboso	0.004	1.202	7	1	1	1	148	16.6	11	0	1	protein me	cytosol;EF	other mole	Pf00828	6157	ENSG000	RPL27A	B2R4B3; I	Cytoplasm	Regulation	15	High	1
TRUE	High	Master Pr	CON_Q3Z	TREMBL:C	0.004	1.201	2	1	1	1	484	54.1	6.16	0	1											0	High	1
TRUE	High	Master Pr	P42766	60S riboso	0.004	1.198	8	1	1	1	123	14.5	11.05	0	1	protein me	cytosol;tra	nucleic ac	Pf00831	11224	ENSG000	RPL35	A8K4V7; I	Cytoplasm	SRP-depe	14	High	1
TRUE	High	Master Pr	P41214	Eukaryotic	0.004	1.192	2	1	1	1	584	64.7	7.65	0	1	cell organi	cytosol;nu	nucleic ac	Pf01253,	1939	ENSG000	EIF2D	hsa:1939;			0	High	1
TRUE	High	Master Pr	Q6NZI2	Caveolae-	0.004	1.187	2	1	1	1	390	43.5	5.6	0	1	RNA meta	plasma m	nucleic ac	Pf15237	284119	ENSG000	CAVIN1	B2RAW7;	Congenita	RHOC GT	5	High	1
TRUE	High	Master Pr	Q9NVU7	Protein SD	0.004	1.181	1	1	1	1	687	79.8	9.25	0	1	transport;c	nucleus		Pf05285,	55153	ENSG000	SDAD1	hsa:55153			0	High	1
